# Single-cell multi-omics reveals circulating B cell heterogeneity across diverse disease contexts

**DOI:** 10.3389/fcell.2026.1897676

**Published:** 2026-08-04

**Authors:** Rui Gan, Yunshan Zhong

**Affiliations:** 1 College of Life Sciences, Beijing Normal University, Beijing, China; 2 Beijing Changping Laboratory, Beijing, China

**Keywords:** atypical B cell, B cell receptor, human immunodeficiency virus, influenza vaccination, melanoma, scBCR-seq, scRNA-seq, systemic lupus erythematosus

## Abstract

**Introduction:**

B cells play essential roles in adaptive immunity by producing antigen-specific antibodies and interacting with T cells. Single-cell RNA and BCR sequencing technologies have enabled high-resolution dissection of B cell heterogeneity across diverse disease contexts. Atypical B cells (ABCs) display complex and critical functions in human immunity, yet their transcriptional heterogeneity, BCR diversity, and context-specific roles remain incompletely understood.

**Methods:**

In this study, we performed an integrative reanalysis of public single-cell RNA sequencing and single-cell B cell receptor (BCR) sequencing data derived from human peripheral blood mononuclear cells (PBMCs) across four distinct clinical contexts, including systemic lupus erythematosus (SLE), immunotherapy-treated melanoma, human immunodeficiency virus (HIV) blip, and age-stratified influenza vaccination. We established a unified analytical framework integrating transcriptomic profiling, BCR embedding-based clustering, and connectivity analysis to systematically characterize circulating B cell transcriptional features, BCR connectivity, and the correlation between transcriptomic and BCR diversity.

**Results:**

Our analysis revealed substantial dynamics in B cell transcriptomics and BCR repertoires across diverse time points and conditions. Notably, ABCs exhibited dynamic fluctuations, strong activation and BCR signaling, and marked transcriptomic diversity in response to autoreactive antigens, immunotherapy, transient viremia, and vaccination, positioning them as critical mediators of B cell responses. By integrating ABCs across all conditions, we identified nine distinct ABC clusters exhibiting specific transcriptional signatures associated with chemotaxis, complement activation, T cell regulation, plasma cell differentiation, or progenitor-like states. These ABC clusters displayed either phenotype-specific or shared distribution patterns, along with varying degrees of B cell activation, BCR signaling, and BCR connectivity across different phenotypes, suggesting divergent functional roles of ABCs.

**Discussion:**

Collectively, our cross-context analysis provides a broad characterization of circulating B cell heterogeneity, BCR diversity, and their associations in diverse conditions, advancing the understanding of ABC functional roles in human diseases. These findings highlight the potential of ABCs as key modulators of B cell responses across autoimmune, cancer, infection, and vaccine contexts.

## Introduction

B cells express specific B cell receptors (BCRs) on their membrane, which enable direct antigen recognition without the involvement of antigen-presenting cells (APCs). Complementarity-determining region 3 (CDR3) plays a decisional role in antigen recognition as the most hypervariable region of the BCR (Zan and Casali, 2016). Upon activation by antigens, B cells proliferate and differentiate into antibody-secreting plasma cells or memory B cells. During this process, B cells undergo dynamic changes in gene expression, triggering the germinal center (GC) reaction, which involves clonal expansion, class switch recombination (CSR), somatic hypermutation (SHM), and the selection of BCR variants with enhanced affinity (LeBien and Tedder, 2008). Upon CSR, B cells shift the isotype of their BCRs from IgM and IgD to IgG, IgA, or IgE, each with distinct effector functions (Bournazos and Ravetch, 2017). The V(D)J recombination of immunoglobulin (Ig) gene segments is now known to generate at least 10^16^ antibody diversities. Upon re-infection by the same pathogen, memory B cells rapidly produce large quantities of specific antibodies to eliminate the antigen. These antibodies enter the circulatory system and tissues, playing a crucial role in humoral immunity (Bonilla and Oettgen, 2010). Furthermore, B cells engage in specific interactions with T cells and present antigens to them, thereby serving as antigen-presenting cells (Cyster and Allen, 2019).

Recently, single-cell RNA sequencing (scRNA-seq) and single-cell B cell receptor sequencing (scBCR-seq) technologies have been widely employed in numerous studies to investigate B cell heterogeneity and function, as well as BCR clonality and diversity, across various disease states. By single-cell transcriptomics analysis, B cells can be classified into several major lineages including naive B (NB) cells, memory B (MB) cells and plasma cells in human blood. Classical memory B cells (MBCs) are generally identified by high expression of *CD27*, whereas atypical B cell (ABC) compartment comprises a subset of memory B cells expressing *TBX21*, *ITGAX* but lacking *CD27*. ABCs have been identified frequently in a range of infectious and autoimmune diseases such as Human Immunodeficiency Virus (HIV) and systemic lupus erythematosus (SLE) as well as expressed autoreactive B cell receptors (Rubtsova et al., 2015), but the functions and markers of ABCs remain elusive and context-dependent (Gao and Cockburn, 2022; Holla et al., 2021; Johnson et al., 2020; Reyes et al., 2023; Sutton et al., 2021). Previous studies have reported that ABCs play distinct roles in human immunities including anergic or exhausted B cells, pre-plasma cells and APCs for T cells, exhibiting complicated biological functions (Gao and Cockburn, 2022; Holla et al., 2021 already used scRNA-seq to characterize heterogeneity in ABC subsets and observed shared transcriptional profiles of ABCs across malaria, HIV, and autoimmune diseases as well as identified ABC subsets based on isotype expression in malaria (Holla et al., 2021). Furthermore, Reyes et al. identified three distinct atypical B cell populations based on expression of ITGAX and CD86 in the context of malaria parasite Plasmodium falciparum by scRNA-seq and flow cytometry (Reyes et al., 2023). In addition, scBCR-seq enables the profiling of BCR clonotypes and CDR3 sequences, facilitating the rapid discovery of large-scale antigen-specific antibodies (Goldstein et al., 2019). Recently, antibody-specific language models have been developed to learn meaningful biological features from massive antibody sequences. For instance, AntiBERTy has been introduced to facilitate the clustering of BCRs with similar antigen specificities, based on the BERT architecture (Devlin et al., 2026; Ruffolo et al., 2021).

Previous studies have generated valuable scRNA-seq and scBCR-seq datasets, including autoantibody-positive SLE (Gao et al., 2022), melanoma with immune-related adverse events (irAEs) following immunotherapy (Çakan et al., 2025), HIV under viral blip (Muir et al., 2026) and influenza vaccination response (Wang et al., 2023). Gao et al., 2024 investigated the role of IL-4R and type I IFN signaling in B cell development and found diminished IL-4R signaling shifts B cell development from a classical memory toward a pathogenic atypical memory phenotype in SLE patients. Çakan et al. focused on how CTLA-4 blockade disrupts peripheral B cell tolerance and found that anti-CTLA-4 (but not anti-PD-1) allowed autoreactive mature naive B cells to accumulate in melanoma patients. Muir et al. investigated the heterogeneity of tissue-like memory (TLM) B cells (CD21^−^CD27^−^), identifying two subpopulations (TLM1 and TLM2) during viral blips in one HIV elite controller. Wang et al. revealed distinct B cell responses to influenza vaccination between young and older responders. While each study provided valuable but context-dependent insights within a single disease context, we propose an integrative analytical framework to systematically characterize circulating B cell heterogeneity by reanalyzing these public single-cell datasets, facilitating cross-context comparisons of B cell response patterns across diverse conditions under a unified analytical pipeline. We aim to investigate the circulating B cell heterogeneity, BCR diversity and their potential associations under various pathological conditions, revealing both disease-specific and shared patterns of B cell dysfunction that were not discernible from any individual study. Notably, by integrating all ABCs across studies, we seek to gain a deeper understanding of ABC heterogeneity and disease specificity across diverse disease types, time points, and age groups. Our study could provide a multifaceted insight of human circulating B cell response in diverse disease contexts.

## Results

### Single-cell analysis reveals B cell heterogeneity and ABC-driven immunity in SLE

**FIGURE 1:**
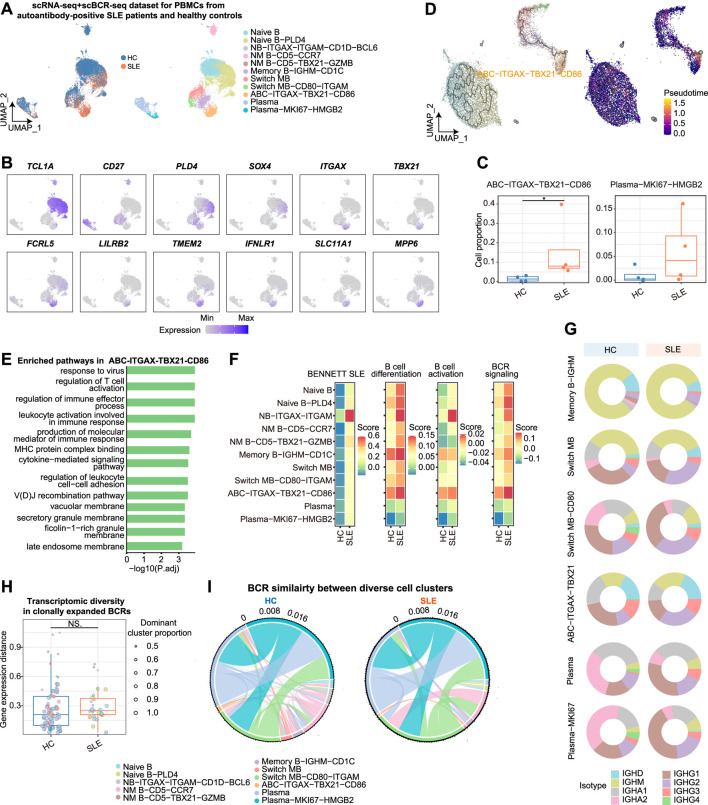
Strong ABC activation and BCR signaling in SLE patients. **(A)** UMAP plots of 12,935 B cells from PBMCs of 3 SLE patients and three healthy controls, with two donors contributing two sample libraries, yielding a total of eight samples, respectively colored by phenotype (left) and cell cluster (right). Cluster identities were defined based on the signature gene expression levels. NB, Naïve **(B)**. NM, Naïve-like memory **(B)**. Switch MB, Class-switched memory **(B)**. ABC, Atypical B cell. **(B)** UMAP plots of normalized gene expression distribution. **(C)** Comparisons of cell cluster proportions between diverse phenotypes. Statistical significance was assessed using Wilcoxon rank-sum test. **(D)** Monocle3 pseudotime UMAP colored by cell cluster (left) or pseudotime (right). Black curves show predicted differentiation trajectories; gray circles indicate trajectory outcomes. **(E)** Top enriched pathways using top 50 upregulated genes in ABCs. **(F)** Distribution of average signature scores for different pathways across diverse cell clusters and phenotypes. **(G)** Distribution of BCR isotype compositions in each cell cluster and phenotype. **(H)** Comparisons of transcriptomic diversity for those clonally expanded BCRs between healthy controls and SLE patients. Each point represents a B cell clone composed of B cells with identical BCR CDR3 sequences. Point color represents the dominant cell cluster in the clone. Point size indicates the cell proportion of the dominant cell cluster in the clone. Statistical significance was assessed using Wilcoxon rank-sum test. **(I)** Chord diagrams showing the average connecting score of BCRs between various cell clusters in BCR networks in diverse phenotypes with a BCR clustering cutoff of 0.08. Each segment represents a cell cluster, with its width proportional to the total connectivity score for the cell cluster. Connecting ribbons indicate the connectivity of BCRs where ribbon width corresponds to the connectivity score and ribbon color indicates the source cell cluster.

Systemic lupus erythematosus (SLE) is a prototypical autoimmune disease characterized by overexpression of granulopoiesis-related and interferon (IFN)-induced genes (Bennett et al., 2003). Here, we depicted the B cell heterogeneity and BCR diversity using paired scRNA-seq and scBCR-seq data from peripheral blood mononuclear cells (PBMCs) derived from six donors including three autoantibody-positive SLE patients and three healthy controls (HC), with two donors contributing two sample libraries, yielding a total of eight samples (Gao et al., 2022). Through single-cell clustering, integration and manually annotation of 12,935 B cells, we totally identified 11 distinct B cell clusters (Figures 1A,B; Supplementary Figure S1A; Supplementary Table S1). Naïve B cells were divided into two clusters based on *PLD4* expression levels, loss-of-function mutations of which are associated with the pathogenesis of SLE (Wang et al., 2025). Compared with naïve B cluster highly expressing *IL4R*, naïve B-PLD4 additionally expressed *NEIL1* and *SUGCT* as well as early progenitor B cell marker *SOX4* (Sun et al., 2013), suggesting that these cells represent a transitional naïve B cell population. Isotype-switched memory B cells were divided into two clusters based on the expression of *IGHM*, *CD80*, *FAS*, *CD86* and *ITGAM*, with Switch MB cluster representing CSR-active memory B cells. We identified an ABC cluster specifically expressing canonical markers including *TBX21*, *ITGAX* and overexpressing previously reported ABC-associated genes such as *CD19*, *FGR*, *FCRL5*, *LILRB1*, *LILRB2*, *SOX5*, *ZEB2*, *CD72*, *CD22* and *CD86* (Gao and Cockburn, 2022; Gao et al., 2024; Holla et al., 2021; Sutton et al., 2021). Plasma cells were classified into two clusters based on *MKI67* expression. In addition, we found three doublet-like cell clusters expressing macrophage or T cell markers. We noticed the *CD5* ^+^ clusters possessed both naïve and memory B cell signatures, suggesting these cells may represent a transitional state between the two differentiation stages. However, the functional significance of this population remains unclear.

Comparative analysis indicated that ABC cluster and proliferating plasma cells were enriched in SLE patients but absent in HCs, in which mature naïve B cluster predominated (Figure 1C; Supplementary Figure S1B). Pseudotime analysis indicated that ABCs probably originated from unswitched memory B cells (Figure 1D). By identifying top upregulated genes in ABCs compared with other memory B cell clusters such as *TMEM2*, *IFNLR1*, *SLC11A1* and *MPP6* (Figure 1B; Supplementary Figure S1C), we found these ABCs were involved in immune response to virus, regulation of T cell activation, cytokine-mediated signaling pathways, cell-cell adhesion and V(D)J recombination by Gene Ontology (GO) analysis (Figure 1E). Notably, the ABCs from SLE patients exhibited higher signature scores of B cell differentiation, activation and BCR signaling than HCs, compared with other memory B cell clusters, which indicated strong B cell response mediated by ABCs in the SLE patients (Figure 1F).

Next, we investigated BCR isotype usage, BCR connectivity, and BCR diversity across diverse cell clusters (Figure 1G; Supplementary Figure S1D-H). Compared with healthy controls, SLE patients showed increased IgG2 usage and decreased IgA2 usage in isotype-switched MBCs, ABCs, and plasma cells, with IgG2 previously reported to correlate with SLE activity indexes (Bruschi et al., 2021). Notably, SLE-derived ABCs also expressed IgD and displayed a restricted yet unbiased isotype profile, lacking both IgA2 and IgG4 usage (Figure 1G). In addition, integrative analysis of BCR and transcriptomics data revealed rapid and strong B cell evolution in SLE patients, with greater transcriptomic diversity among clonally expanded BCRs (Figure 1H). Through BCR CDR3 embedding and clustering analysis using a cosine distance cutoff of 0.08 determined by gradient evaluation (Supplementary Figures S1G, 2 and Methods), we observed a slightly higher ABC BCR repertoire diversity in SLE than healthy samples (Supplementary Figure S1H). We further observed strong BCR connectivity between switched MBCs and plasma cells but very limited BCR connectivity between ABCs with Naïve B or Switch MB clusters (Figure 1I). Overall our results revealed strong B cell response in the SLE patients.

### Dynamics of B cells across diverse time points for melanoma patients during immunotherapy

Anti-CTLA-4 and anti-PD-1 therapy frequently induce immune-related adverse events (irAEs) during clinical treatment (Yin et al., 2023). Previous studies have implicated the potential role of ABCs in the pathogenesis of irAEs (Das et al., 2018; Dhodapkar et al., 2023). Here, we investigated the dynamics of B cell composition and BCR repertoires in the PBMCs from five melanoma patients with irAEs (such as thyroiditis and hypophysis) following immunotherapy across diverse time points at baseline (d0, before treatment), 6 weeks (6w with anti-CTLA-4 and anti-PD-1 combination therapy) and 7 months (7 m with anti-PD-1) (Çakan et al., 2025).

**FIGURE 2:**
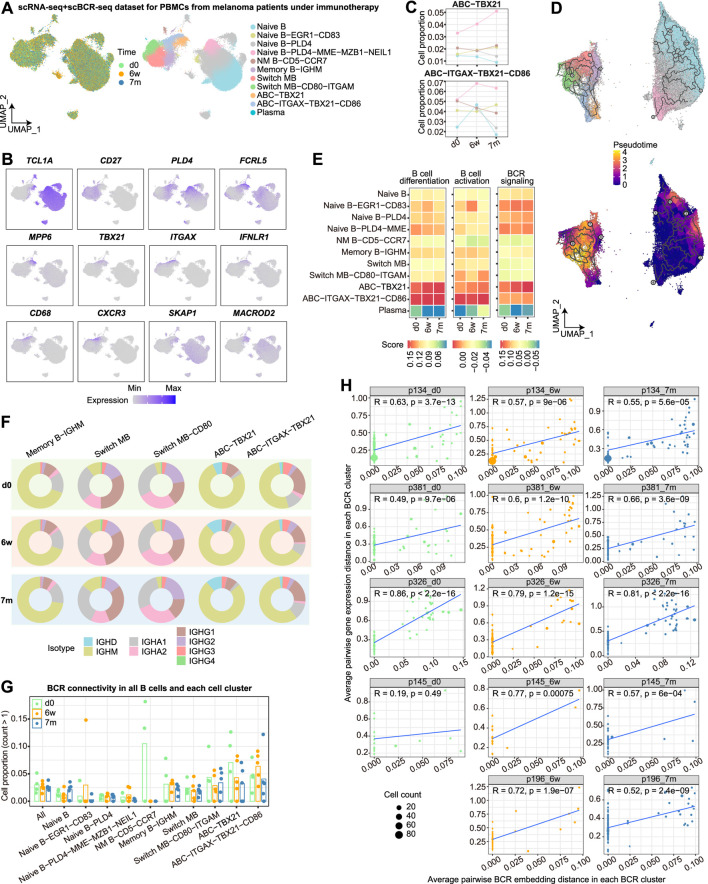
Dynamics of B cells in melanoma patients during immunotherapy. **(A)** UMAP plots of 56,412 B cells from PBMCs of 5 melanoma patients, respectively colored by time point (left) and cell cluster (right). D0, before treatment. 6w, 6 weeks 7m, 7 months. **(B)** UMAP plots of normalized gene expression distribution. **(C)** Comparisons of cell cluster proportions between diverse time points. **(D)** Monocle3 pseudotime UMAP colored by cell cluster (top) or pseudotime (bottom). Black curves show predicted differentiation trajectories; gray circles indicate trajectory outcomes. **(E)** Distribution of average signature scores for different pathways across diverse cell clusters and phenotypes. **(F)** Distribution of BCR isotype compositions in each cell cluster and time point. **(G)** Distribution of BCR connectivity in all B cells and diverse cell clusters across different time points with a BCR clustering cutoff of 0.06. Bar plots showing the average BCR connectivity (the proportion of cells clustered with other cells in a BCR cluster in each sample) across diverse time points. Point color indicates the sample time point. **(H)** Pearson’s correlation between BCR diversity (average pairwise BCR CDR3 embedding distance) with transcriptomic diversity (average pairwise gene expression distance) across all BCR clusters (at least 2 cells) in each sample with a BCR clustering cutoff of 0.1. Each point represents a BCR cluster. Point size indicates the number of cells in the BCR cluster. Pearson’s correlation coefficient and *P* value are indicated on the top of each panel.

Single-cell transcriptomic analysis revealed 11 distinct B cell clusters containing 56,412 cells (Figures 2A,B; Supplementary Figure S3A; Supplementary Table S1). We identified a transitional B cell cluster expressing *MME*, *MZB1*, *CD38*, *NEIL1* and *PLD4*. As identified in the SLE data, we observed a *CD5* ^+^ cluster overexpressing *CCR7* that exhibited both naïve and memory B cell signatures. Besides the ABC-ITGAX-TBX21-CD86 cluster, we additionally identified a distinct ABC cluster expressing *TBX21* but lacking *ITGAX* and *CD86*, which both overexpressed ABC-related genes such as *FCRL5*, *FGR* and *SOX5*. Differential gene expression analysis revealed that ABC-TBX21 cluster highly expressed naïve B cell-related markers such as *TCL1A*, *IGHD*, *PCDH9*, *SKAP1, MACROD2, BCL7A* and *BACH2* while ABC-ITGAX-TBX21-CD86 highly expressed myeloid and inhibitory receptors such as *CD68*, *CD1C*, *CXCR3*, *TFEC*, *SIGLEC6* and *SIGLEC10* (Supplementary Figure S3B). GO analysis indicated that ABC-ITGAX-TBX21-CD86 cluster was specifically enriched in pathways related to regulation of inflammatory response, myeloid activation and cytokine-mediated signaling (Supplementary Figure S3C). The abundance of this ABC cluster peaked at 6 weeks and declined at 7 months, similar to the trend of autoreactive naive B cells as reported by the authors (Çakan et al., 2025) (Figure 2C; Supplementary Figure S3D). Pseudotime analysis suggested a potential topological trajectory of ABC-TBX21 cluster towards either ABC-ITGAX-TBX21-CD86 cluster or switched memory B cells (Figure 2D). Moreover, ABC-TBX21 cluster exhibited stronger BCR signaling signatures while ABC-ITGAX-TBX21-CD86 cluster showed higher activation scores following treatment, which both displayed remarkably active differentiation scores than Naïve B cells (Figure 2E).

Furthermore, we investigated the dynamics of BCR repertoires during immunotherapy. The composition of BCR isotype usage remained stable across diverse time points (Figure 2F). In contrast to ABCs observed in the SLE patients, most ABCs expressed IgM in the PBMCs of melanoma. ABC-ITGAX-TBX21-CD86 cluster showed higher frequencies of IgG1 or IgA1 usage than ABC-TBX21 cluster. We further observed very low B cell clonal expansion rate of less than 5% in all patients (Supplementary Figure S3E). For those clonally expanded B cells, we observed an increasing tendency of transcriptomic diversity from d0 to 7m, revealing stronger B cell response during immunotherapy (Supplementary Figure S3F). BCR embedding-based clustering analysis with a cosine distance cutoff of 0.06 revealed that ABCs exhibited stronger BCR connectivity than other B cell clusters, as measured by the proportion of cells from multicellular BCR clusters (Supplementary Figure S4). Within ABCs, ABC-TBX21 cluster peaked at d0 while ABC-ITGAX-TBX21-CD86 peaked at 6w (Figure 2G). To explore the link between BCR sequence and transcriptomic profiles, we correlated intra-cluster BCR embedding distances with gene expression distances across a range of cosine distance cutoffs. Using relatively inclusive cutoffs that yielded more BCR clusters composed of at least two cells, we observed a clear positive correlation between transcriptomic and CDR3 embedding distances, indicating that B cells with similar BCRs tend to share similar transcriptional states (Figure 2H; Supplementary Figure S5). Furthermore, comparative analysis of BCR repertoires revealed varying levels of BCR connectivity between various cell clusters and across time points (Supplementary Figure S3G, 6). We observed two ABC clusters shared similar CDR3s with each other and unswitched memory B cells at each time point (Supplementary Figure S3G). Comparative analysis of cross-time BCR repertoires in each patient displayed only a minor fraction of similar BCRs were shared across various time points, which exhibited great heterogeneity among diverse patients (Supplementary Figure S6). Altogether, our analyses depicted the great dynamic changes of circulating B cell transcriptomics and BCR compositions as well as their potential associations during response to immunotherapy in melanoma patients.

### A case study of B cell responses to transient HIV viremia in a single elite controller

HIV infection induces B cell dysfunction and excessive accumulation of ABCs in the blood of HIV patients (Moir and Fauci, 2009; Moir et al., 2008; Muir et al., 2026). Here, we aimed to explore the transcriptomic and BCR dynamic changes of B cells during transient HIV viremia by integrating paired scRNA-seq and scBCR-seq data from the PBMCs of an HIV elite controller, with three replicates per time point collected before, during and after viral blip (Muir et al., 2026).

**FIGURE 3:**
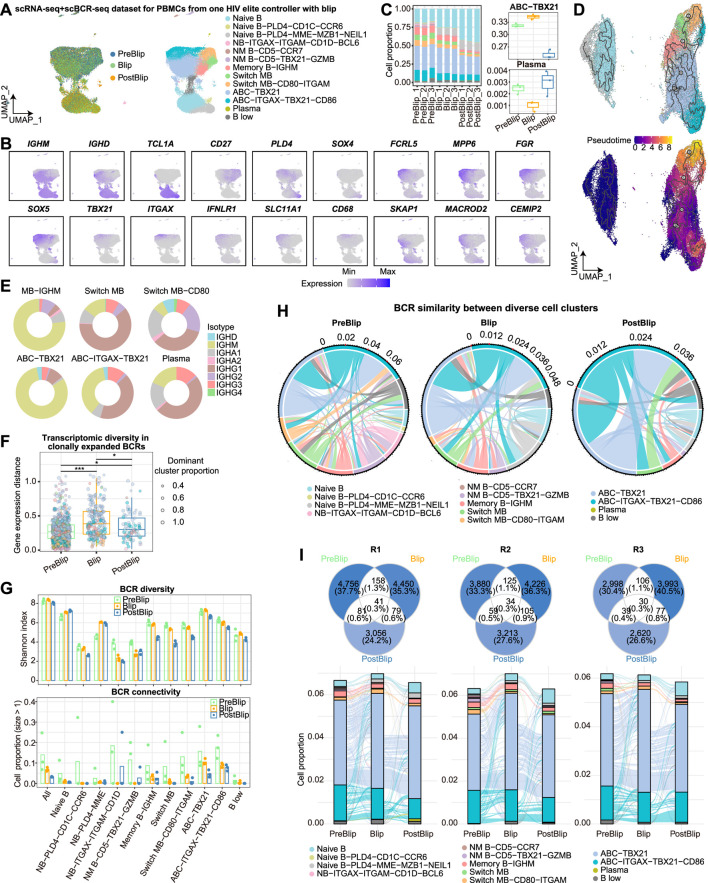
Dynamics of B cell response to HIV viral blip in one elite controller. **(A)** UMAP plots of 36,888 B cells from PBMCs of one HIV elite controller with three replicates at each time point, respectively colored by time point (left) and cell cluster (right). **(B)** UMAP plots of normalized gene expression distribution. **(C)** The left panel shows the cell cluster distribution in each sample. The right panel shows the comparisons of cell cluster proportions between diverse time points. **(D)** Monocle3 pseudotime UMAP colored by cell cluster (top) or pseudotime (bottom). Black curves show predicted differentiation trajectories; gray circles indicate trajectory outcomes. **(E)** Distribution of BCR isotype compositions in each cell cluster and phenotype. **(F)** Comparisons of transcriptomic diversity for those clonally expanded BCRs across diverse time points. Each point represents a B cell clone composed of B cells with identical BCR CDR3 sequences. Point color represents the dominant cell cluster and point size indicates the dominant cell cluster proportion in the clone. Statistical significance was assessed using Wilcoxon rank-sum tests. **(G)** Distribution of BCR diversity and BCR connectivity in all B cells and diverse cell clusters across different time points with a BCR clustering cutoff of 0.1. **(H)** Chord diagrams showing the average connectivity score of BCRs between various cell clusters in BCR networks in diverse phenotypes with a BCR clustering cutoff of 0.1. **(I)** The top panel displays the intersections of BCR clusters across different time points for each replicate group with a BCR clustering cutoff of 0.1. Overlapping BCR cluster numbers and proportions are indicated in the Venn diagrams. The bottom Sankey plots visualize the cell cluster distribution of connected BCRs across time points. Ribbons connect cells from two time points that share the same BCR cluster, with ribbon color denoting the B cell cluster. The y-axis represents the aggregated proportion of connected cells from all dominant clusters at each time point per BCR cluster.

In this case study, we totally identified 13 distinct B cell clusters among 36,888 B cells (Figure 3A; Supplementary Table S1). ABCs were classified into two clusters based on the expression levels of *ITGAX* and *TBX21*, with ABC-TBX21 cluster lowly expressing *TBX21* and lacking *ITGAX* as observed in the melanoma data (Figure 3B; Supplementary Figure S7A). Throughout the viral blip, we observed four distinct dynamic trends in this elite controller. ABC-TBX21 cluster specifically exhibited a different tendency of a minor increase followed by a sharp decline, while the ABC-ITGAX-TBX21-CD86 cluster and other memory B cells demonstrated a persistent decrease. Comparatively, plasma cells first decreased and then increased sharply, while most naïve B cells showed a continuous increase (Figure 3C; Supplementary Figure S7B). Pseudotime analysis showed three potential transcriptional transitions from ABC-TBX21 to ABC-ITGAX-TBX21-CD86, from ABC-ITGAX-TBX21-CD86 to class-switched MBCs, and from unswitched memory B cells to ABC-TBX21 (Figure 3D). While ABC-TBX21 cluster highly expressed naïve B-enriched genes, ABC-TGAX-TBX21-CD86 overexpressed metabolism, neurological or immunity-related genes such as *GPR137B*, *SYT1*, *ENC1* and *TOX*, *CD68*, *IFNLR1* (Gan et al., 2019; Godsk et al., 2025; Mohseni Ahooyi et al., 2019) (Supplementary Figure S7C). GO analysis showed ABC-TGAX-TBX21-CD86 cluster was enriched in pathways such as antigen presentation, cytokine production and cell adhesion (Supplementary Figure S7D). Additionally, we observed ABCs and other naïve B clusters displayed higher differentiation, activation and BCR signaling scores before viral blip compared with other time points (Supplementary Figure S7E). Analysis of BCR repertoires showed that ABC-TBX21 cluster preferred IgM usage while ABC-TGAX-TBX21-CD86 cluster preferred IgG1 usage (Figure 3E).

Next, we observed that the strength of BCR clonal expansion progressively diminished during viral blip (Supplementary Figure S7F). The transcriptomic diversity of these clonally expanded B cells increased during the blip but decreased thereafter. Notably, ABCs exhibited high cellular heterogeneity in each clone during the viral blip, suggesting a strong ABC-related response induced by HIV viral blip (Figure 3F). Embedding-based BCR clustering analysis (cosine distance<0.1) further showed that pre-blip BCRs exhibited the strongest BCR connectivity, which progressively decreased afterward (Figure 3G; Supplementary Figure S8). Correlation analysis further revealed a tighter coupling of BCR diversity and B cell transcriptomic diversity during or after the blip than before the blip, across a range of clustering cutoffs (Supplementary Figure S9A-D). We further observed a stronger BCR connectivity across diverse cell clusters at the pre-blip and blip time points, with ABC-dominated BCR connectivity persisting after the blip (Figure 3H). Cross-time BCR clustering analysis revealed only a small fraction (∼0.06) of ABC-mediated BCR connectivity occurred between various time points (Figure 3I; Supplementary Figure S9E).

Altogether, our results indicated that B cell activation and evolution were strongest before blip and reached the highest cellular heterogeneity during the blip, and subsequently declined after the blip in the elite controller. These findings suggest ABC-mediated B cell dysfunction in the PBMCs of HIV patients during transient viremia, pending validation in larger cohorts.

### Distinct B cell responses to influenza vaccine between the young and older adults

**FIGURE 4:**
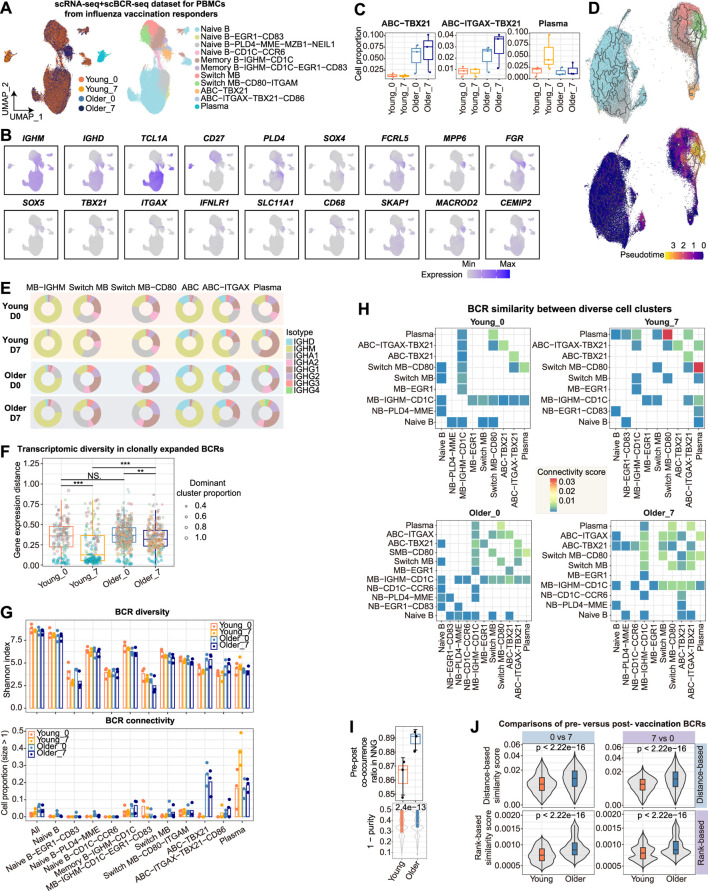
Distinct B cell responses to influenza vaccination in young and older responders. **(A)** UMAP plots of 71,031 B cells from PBMCs of three young and three older influenza vaccination responders colored by time point (left) and cell cluster (right). 0 and 7 respectively represents pre-vaccination and post-vaccination at 7 days. **(B)** UMAP plots of normalized gene expression distribution. **(C)** Comparisons of cell cluster proportions between diverse phenotypes. **(D)** Monocle3 pseudotime UMAP colored by cell cluster (top) or pseudotime (bottom). Black curves show predicted differentiation trajectories; gray circles indicate trajectory outcomes. **(E)** Distribution of BCR isotype compositions in each cell cluster and phenotype. **(F)** Comparisons of transcriptomic diversity for those clonally expanded BCRs between diverse time points. Each point represents a B cell clone composed of B cells with identical BCR CDR3 sequences. Point color represents the dominant cell cluster and point size indicates the dominant cell cluster proportion in the clone. Statistical significance was assessed using Wilcoxon rank-sum test. **(G)** Distribution of BCR diversity and BCR connectivity in all B cells and diverse cell clusters across different phenotypes with a BCR clustering cutoff of 0.06. **(H)** Heatmaps showing the average connectivity score of BCRs between various cell clusters in diverse phenotypes with a BCR clustering cutoff of 0.06. **(I)** The top panel shows the proportion of BCR clusters containing both pre- and post-vaccination BCRs based on nearest-neighbor graphs in each responder. The bottom panel shows the distribution of 1-purity in these pre-post BCR occurrence clusters. Purity represents the proportion of BCRs originating from the dominant time point within a BCR cluster. **(J)** Comparisons of BCR similarity among pre- versus post-vaccination B cells between young and older responders. 0 vs. 7 represents the average BCR similarity of each pre-vaccination BCR compared with all post-vaccination BCRs. 7 vs. 0 represents the average BCR similarity of each post-vaccination BCR compared with all pre-vaccination BCRs. Statistical significance was assessed using Wilcoxon rank-sum tests.

Among patients with infectious diseases, older adults exhibit impaired responses to vaccination in contrast to young individuals (Czaja et al., 2019; Goodwin et al., 2006). To investigate age-related differences in B cell responses to vaccination, we performed integrative analysis of paired scRNA-seq and scBCR-seq data from PBMCs of three young and three older influenza responders before vaccination (D0) and 7 days post-vaccination (D7) (Wang et al., 2023). In this study, we totally identified 11 distinct B cell clusters from 71,031 cells based on the expression levels of previously established canonical markers (Figures 4A,B; Supplementary Figure S10A; Supplementary Table S1). Notably, ABCs were significantly enriched in older responders compared to young responders. Plasma cells showed a marked increase after vaccination in the young responders but remained stable in the older responders (Figure 4C; Supplementary Figure S10B). Pseudotime analysis suggested a potential topological trajectory from ABC-TBX21 or unswitched MBCs to ABC-ITGAX-TBX21 and then to class-switched MBCs (Figure 4D). Compared with other memory B cells, ABC-TBX21 cluster upregulated immune-regulated genes such as *LILRA4*, *TCL1A*, *IGKV4-1*, *MACROD2* and *PCDH9*, which was enriched in pathways such as regulation of inflammatory response, response to calcium ion, muscle hypertrophy and phagocytosis. Comparatively, ABC-ITGAX-TBX21-CD86 highly expressed genes such as *ITGAX*, *TBX21*, *GPR137B*, *S100A11*, *ADGRE5* and *TNFRSF1B*, and was enriched in pathways including integrin-mediated cell adhesion, macrophage activation, respiratory burst, cytokine production (Supplementary Figure S10C,D). Both ABC clusters exhibited the highest B cell differentiation and BCR signaling scores, with the ABC-ITGAX-TBX21-CD86 cluster being the most activated among all cell clusters (Supplementary Figure S10E). Compared with young responders, the older responders displayed more enriched IgG2 usage. Plasma cells showed an increased IgG1 usage after vaccination (Figure 4E).

We further observed that young responders showed a lower overall BCR clonal expansion rate than the older responders (Supplementary Figure S11A). Notably, the young responders exhibited a stronger clonal expansion of plasma cells, whereas the older responders showed significant clonal expansion of ABCs particularly the ABC-TBX21 cluster (16.7%–33.4%) (Supplementary Figure S11B). We found these clonally expanded B cells exhibited higher transcriptomic diversity before vaccination, followed by sharper decline after vaccination in the young responders than in the older responders (Figure 4F). Embedding-based BCR clustering analysis (cosine distance<0.06) further indicated distinct B cell response to vaccination with higher post-vaccination BCR connectivity of ABC-TBX21 in the older responders but of plasma cells in the young responders (Figure 4G; Supplementary Figure S12). We found plasma cells showed a stronger BCR connectivity with *CD80* ^+^ class-switched memory B cells and ABC-ITGAX-TBX21-CD86 after vaccination in the young responders, whereas the older responders exhibited stronger BCR connectivity of B cells between ABCs and other memory B cells (Figure 4H). We next examined the correlation between BCR diversity and transcriptomic diversity across a range of clustering cutoffs. This correlation was higher in young responders before vaccination, whereas a slightly weaker association was observed after vaccination in both young and older responders (Supplementary Figure S13). Furthermore, young responders exhibited greater changes in BCR composition in response to vaccination compared to older responders, who showed higher co-occurrence ratios and similarity scores between pre- and post-vaccination BCRs (Figures 4I,J; Supplementary Figure S11C,D). Cross-time BCR clustering analysis showed that young responders were characterized by pre- versus post-vaccination BCR connectivity of mature naïve B and unswitched memory B cells but the older responders showed more enrichment of ABC-related BCR connectivity (Supplementary Figure S11E).

Taking together, our results revealed distinct patterns of circulating B cell response to influenza vaccination in young and older responders. Young responders were characterized by great BCR dynamics and strong clonal expansion of plasma cells, supporting concordant antibody-secreting functions in defense of virus. Comparatively, older responders exhibited large accumulation of ABCs and a weaker B cell response, displaying minimally altered cellular and BCR compositions in response to vaccination.

### High heterogeneity of ABCs in human PBMCs across diverse conditions

**FIGURE 5:**
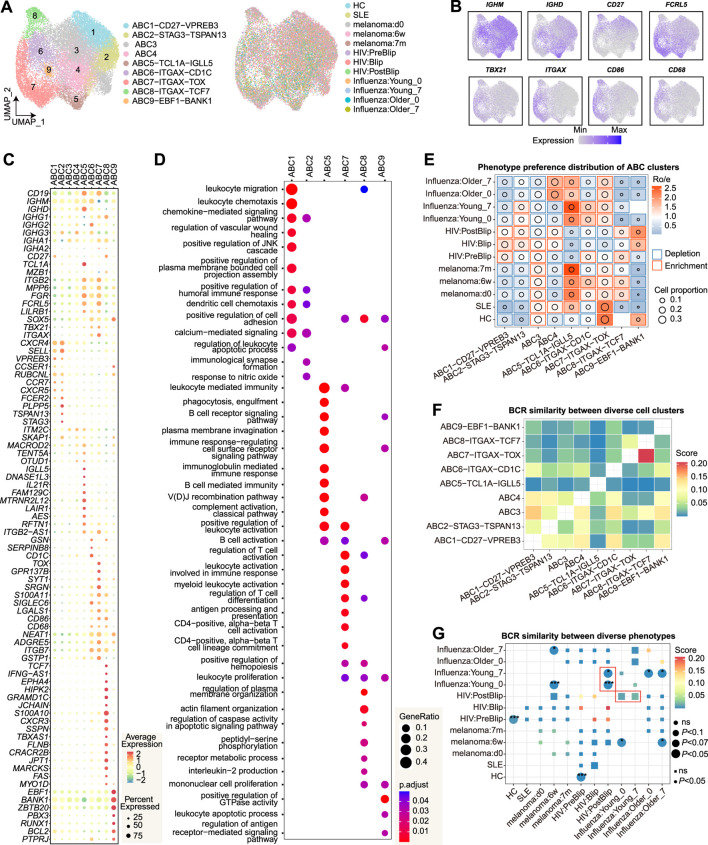
Heterogeneity of ABCs across diverse disease contexts. **(A)** UMAP plots of 23,373 ABCs, respectively colored by cell cluster (left) and phenotype (right). **(B)** UMAP plots of normalized gene expression distribution. **(C)** Dot plot showing the expression of top significant upregulated genes in each cell cluster. Point size indicates the percentage of cells expressing the gene in the cell cluster. Point color indicates the scaled gene expression distribution across diverse phenotypes. **(D)** Top enriched pathways using top 100 upregulated genes in each ABC cluster. Point size indicates the proportion of genes involved with the pathway in all genes for the cell cluster. Point color indicates the adjusted *P* value calculated using enrichGO function. **(E)** Phenotype preference of each ABC cluster evaluated by the Ro/e index. Point size indicates the proportion of cells for the cell cluster in each phenotype. Red (Ro/e > 1) represents enrichment of the cluster in the phenotype. **(F)** Heatmap showing the connectivity score of BCRs between various cell clusters with a BCR clustering cutoff of 0.1. **(G)** Bubble plot showing the connectivity score of BCRs between various phenotypes with a BCR clustering cutoff of 0.1. Point shape represents the significance of BCR connectivity between two phenotypes by within-donor permutation tests. Point size indicates the *P* value by the permutation tests. Significance level was denoted in the corresponding point (*P < 0.05, **P < 0.01, ***P < 0.001).

Our cross-disease analyses highlighted the important role of ABCs in B cell responses. To further characterize their heterogeneity, we integrated 23,373 ABCs from diverse disease contexts and systematically profiled their cellular states, putative functions, and BCR connectivity. Following batch effect correction and initial clustering at a resolution of 1.0, we manually merged highly similar clusters based on canonical marker expression and the correlation of top upregulated genes, ultimately yielding nine distinct ABC clusters (Figures 5A,B; Supplementary Figure S14; Supplementary Table S2). To evaluate batch effect correction performance, we computed the local Inverse Simpson’s Index (LISI) for each cell at both the study and donor levels. Mean LISI scores were 2.16 (out of 4 studies) and 4.32 (out of 18 donors), indicating effective mixing without overcorrection (Supplementary Figure S15). To evaluate the stability of the nine ABC clusters, we performed 100 iterations of cell-level random 80/20 train-test split and used Seurat’s label transfer to predict test cell labels. Mean accuracy and F1 scores were 0.774 and 0.767, with all clusters showing F1 scores >0.7 except for ABC4. ABC7 achieved the highest F1 (0.824), whereas ABC4 showed the lowest (0.629), indicating ABC4 probably represented a phenotype-specific or transitional subset that was less robust to dataset shift (Supplementary Figure S15B). Furthermore, leave-one-dataset-out cross-validation yielded prediction accuracies of 0.61 (SLE), 0.75 (melanoma), 0.73 (HIV), and 0.64 (influenza). The reduced stability of ABC4 and modest cross-dataset performance of SLE and influenza datasets highlight the substantial ABC heterogeneity across diverse cohorts and clinical contexts, underscoring the need for external validation in larger independent cohorts to confirm the observed ABC subsets (Supplementary Figure S15C).

Correlation and comparative analysis of the top 50 upregulated genes per cluster (log_2_ fold change>1) revealed substantial cell heterogeneity in ABCs, with most clusters expressing specific sets of signature genes. Notably, ABC3 and ABC4 exhibiting gene expression profiles that were highly similar to those of other clusters, probably representing transition states (Supplementary Figure S15D,E). ABC1 and ABC8 highly expressed *CD27* in contrast to other clusters. ABC1-CD27-VPREB3 exhibited extremely similar expression profiles with ABC2-STAG3-TSPAN13, with high expression of chemokines and B cell differentiation markers such as *CXCR4*, *CCR7*, *CXCR5*, *SELL*, *VPREB3* and *FCER2* while ABC2 additionally overexpressed differentiation-related factor *PLPP5* (Trezise et al., 2018) and pro-apoptosis marker *TSPAN13* (Zhang et al., 2024), which were both enriched in pathways such as chemokine-mediated signaling, dendritic cell chemotaxis and calcium-mediated signaling (Figures 5C,D). ABC5-TCL1A-IGLL5 mainly participated in pathways such as BCR signaling, B cell activation, V(D)J recombination, phagocytosis and complement activation, characterized by high expression of *TBX21*, *CD19*, *IGHD*, *TCL1A*, *FAM129C*, *IGLL5*, *IL21R*, *DNASE1L3*, *LAIR1*, *RFTN1*, *AES*. Compared with other cell clusters, ABC6-ITGAX-CD1C, ABC7-ITGAX-TOX and ABC8-ITGAX-TCF7 all highly expressed ABC canonical markers such as *ITGAX*, *TBX21*, *CD68*, *CD86*, *S100A11*, *SIGLEC6*, *LGALS1* and *ITGB7*. ABC6 and ABC7 exhibited highly similar transcriptional profiles. Specifically, ABC6 overexpressed *IGHM*, *CD1C* and *SERPINB8* while ABC7 downregulated *IGHM* and overexpressed *TOX* and *GPR137B*, *SRGN* and *SYT1*, mainly enriched in pathways of macrophage/T cell activation and regulation of hemopoiesis. Comparatively, ABC8 exhibited heightened expression of T cell and plasma cell development markers such as *TCF7*, *MZB1*, *JCHAIN*, *CXCR3*, *FAS* as well as actin filament organization markers such as *FLNB*, *MYO1D, JPT1*, *SSPN*, *S100A10*. ABC9-EBF1-BANK1 overexpressed canonical B-cell development markers such as *EBF1*, *BANK1*, *ZBTB20*, *PBX3*, *RUNX1*, *BCL2* and *PTPRJ*. Among all ABC clusters, ABC9 exhibited the highest B cell activation and BCR signaling scores (Supplementary Figure S16A). Most ABC clusters exhibited the most frequent IgM isotype usage while ABC7 and ABC8 preferred IgG1 usage (Supplementary Figure S16B).

Furthermore, evaluation of enrichment of ABC clusters with Ro/e analysis (Zhang et al., 2018) revealed both phenotype-specific and phenotype-shared circulating ABC subsets (Figure 5E). Several ABC clusters exhibited strong disease preferences. ABC7 was markedly enriched in SLE patients, while ABC4 predominated in older influenza vaccine responders. ABC9 showed a highly specific enrichment in HIV patients with viral blip. ABC5 displayed a strong distribution preference across both melanoma and influenza-associated subjects but depleted in the HIV elite controller. Comparatively, other ABC clusters showed more moderate or shared enrichment patterns. ABC6 and ABC7 were slightly enriched in both melanoma and influenza vaccination responders. ABC1 and ABC2 were modestly enriched in HIV patients. Additionally, ABC2 exhibited a slight preference for young influenza vaccine responders. ABC8 showed a modest preference for melanoma and HIV patients. In contrast, ABC3 showed no phenotype-specific enrichment and was distributed broadly across all conditions. Collectively, we identified diverse phenotype-enriched ABC clusters.

We next evaluated BCR similarity across diverse ABC clusters and phenotypes by embedding-based BCR clustering analysis with a clustering cutoff of 0.1 (Supplementary Figure S17). We noticed that ABC1, ABC2, ABC3, ABC4, and ABC6 shared similar BCRs with each other, whereas ABC5, ABC7, ABC8 and ABC9 exhibited specific BCR features. Notably, ABC8 shared the most similar BCRs (>20%) with ABC7 (Figure 5F). Furthermore, we observed stronger BCR connectivity in HIV and older influenza vaccination responders compared with other disease types. ABC8 exhibited strong BCR connectivity in HIV patients and ABC9 specifically displayed strongest BCR connectivity in the post-blip HIV patient (Supplementary Figure S16C). In addition, we observed disease-specific BCR connectivity of ABCs, whereas diverse phenotypes exhibited extremely weak BCR connectivity, likely due to the high specificity of BCR repertoires among different individuals (Figure 5G). Notably, we found the post-influenza vaccination responders exhibited a BCR connection score of 2% with the post-blip HIV patient, suggesting embedding-based BCR sequence similarity of uncertain antigenic relevance. To assess the statistical significance of this cross-context BCR connectivity, we performed within-donor permutation tests with 1,000 random shuffles of BCR phenotype labels per donor. *P* value was defined as the proportion of permuted connectivity scores that exceeded the observed score. This analysis revealed significant BCR connectivity between post-blip HIV patients and young influenza vaccine responders in two directional comparisons: pre-vaccination vs. post-blip HIV (*P* < 0.001) and post-vaccination vs. post-blip HIV (*P* = 0.01) (Figure 5G; Supplementary Figure S16D).

In conclusion, we characterized the transcriptomic and phenotypic heterogeneity of human circulating ABCs across diverse disease types, time points and age groups. Our analyses identified candidate biomarkers of distinct ABC clusters and suggested their potential functional roles in the humoral immune response.

## Discussion

In this study, we depict the B cell heterogeneity and BCR connectivity as well as their potential associations in human PBMCs across diverse pathogenic diseases including SLE, melanoma, HIV and influenza under different conditions. Our integrative analyses reveal the significance and distinct response patterns of PBMC-derived ABCs in autoimmune, immunotherapy, viral blip and vaccination associated responses.

First, we propose an analytical framework that integrates single-cell transcriptomic and BCR data to characterize circulating B cell distribution patterns and states across various disease contexts across multiple dimensions. We characterized B cell transcriptional features, B cell compositions as well as BCR isotype, BCR diversity and connectivity. We further assessed the correlation of transcriptomic diversity with BCR diversity, and BCR dynamics across diverse cell clusters, phenotypes and time points. Among all disease contexts, we could respectively observe marked B cell dynamics between diverse conditions. In SLE dataset, we observed significant elevation of ABCs and proliferated plasma cells as well as decline of mature naïve B cells in SLE patients compared with HC controls. SLE patients exhibited hyperactivation of ABCs and strong BCR signaling. In order to evaluate the strength of B cell response, we defined transcriptomic diversity of BCRs as the average pairwise gene expression distance. An elevated transcriptomic diversity of clonally expanded BCRs was observed in SLE patients, indicating the potential dysregulated B cell response to autoantigens. In melanoma dataset, we found the abundance of *ITGAX* ^+^ *TBX21* ^+^ ABCs mirrored the trend of the autoreactive naïve B cells from the original paper, showing a characteristic rise followed by a fall. We found the transcriptomic diversity of these clonally expanded BCRs gradually increased during melanoma immunotherapy process. In HIV dataset, the transcriptomic diversity and abundance of *TBX21* ^+^ ABCs initially increased and subsequently decreased across the pre-, during and post-viral blip phases. In influenza dataset, the transcriptomic diversity significantly decreased after vaccination especially in the young responders with strong clonal expansion of plasma cells. BCR clustering analysis revealed that BCR embedding distance and transcriptomic distance showed strong associations across multicellular BCR clusters, suggesting that B cells with more similar BCRs probably display more similar transcriptional features during clonal evolution. We also observed the strong associations between transcriptomic and BCR diversity in HIV and influenza patients.

Especially, we depict the transcriptional states and BCR diversity of human circulating ABC clusters. At a coarse-grained level, ABCs can be classified into two subsets. Cluster1 highly expressed *ITGAX*, *TBX21* together with *CD86* and *CD68* while cluster2 lowly expressed *TBX21* with lack of *ITGAX*. At a fine-grained level, ABCs can be grouped into nine distinct clusters with diverse transcriptional features. ABC1 (*CD27* ^+^ *ITGAX* ^−^ *TBX21* ^low^) and ABC2 (*CD27* ^−^ *ITGAX* ^-^ *TBX21* ^low^) are involved with cell chemotaxis with high expression of *CXCR4* and *CCR7*. ABC3 and ABC4 exhibits as transient cell states with no obvious biomarkers, in which ABC4 shows higher expression of canonical ABC markers. ABC5 (*CD27* ^low^ *ITGAX* ^−^ *TBX21* ^+^) is strongly associated with BCR signaling and complement activation, overexpressing B-cell development markers such as *CD19*, *IGHD*, *TCL1A*, *FAM129C* as well as tumor-associated gene such as *IGLL5* (Chen et al., 2026). ABC6 (*CD27* ^low^ *ITGAX* ^++^ *TBX21* ^++^) highly expressed *IGHM*, *IGHG2*, *CD1C* and *SERPINB8*, indicating its potential role in antigen presentation. ABC7 (*CD27* ^−^ *ITGAX* ^++^ *TBX21* ^++^) and ABC8 (*CD27* ^+^ *ITGAX* ^+^ *TBX21* ^+^) overexpressed T-cell regulation markers such as *TOX* or *TCF7* and *CXCR3* (Pereira et al., 2017). In addition, ABC8 also exclusively expressed plasma cell markers such as *JCHAIN* and *MZB1* but lowly expressed *IGHD* and *IGHM* (Reyes et al., 2023), indicating this cluster probably represented a transitional state towards plasma cells. ABC9 (*CD27* ^−^ *ITGAX* ^low^ *TBX21* ^-^) highly expressed early B-cell development markers such as *EBF1*, *BANK1*, *PBX3* and *RUNX1*, exhibiting as a putative progenitor-like B cell state. Overall, our results systematically depict transcriptional programs of human circulating ABCs across multiple disease conditions, at a granularity that enables the identification of previously unappreciated ABC subsets and provides a foundation for future functional validation.

However, several limitations of this study should be acknowledged. First, the relatively limited cell numbers, particularly the modest count of ABCs from healthy controls, may reduce the statistical power and generalizability of the HC-related comparisons. Second, the HIV dataset includes only a single donor, which restricts our ability to assess inter-individual variability in the context of viral blip responses. Third, *CD5* ^+^ clusters (*TCL1A* ^+^ *CD27* ^+^) detected in three distinct studies also co-expressed T-cell lineage markers in partial datasets, which may introduce technical noise. While we included these clusters in certain analyses to maintain the full biological spectrum and avoid over-filtering, we acknowledge that the corresponding results require further validation in independent cohorts. Fourth, although BCR embedding-based connectivity provides a powerful approach to capture BCR sequence similarity, we caution against interpreting such connectivity as direct evidence of clonal expansion. Rather, it reflects CDR3 sequence similarity, which may arise from convergent recombination events or shared structural features. Fifth, the moderate leave-one-dataset-out prediction accuracies (0.61–0.75) indicate that cross-dataset generalizability of the integrated ABC clusters remains limited due to substantial biological heterogeneity. In addition, the trajectory inferences and functional annotations of these identified ABC clusters rely predominantly on transcriptional features. These considerations underscore the need for independent cohort validation and functional experiments to confirm our findings. Finally, our findings only reflect the circulating B cell heterogeneity rather than tissue-resident or lymph node compartments. Whether the ABC subsets and transcriptional programs identified here are also present at sites of inflammation, such as affected tissues in SLE or the tumor microenvironment in melanoma, remains unknown.

Collectively, our study improves the understanding of circulating B cell heterogeneity and distinct B cell responses across various disease contexts related with autoimmune, cancer, virus infection and vaccination. We highlight the potential roles of ABCs in B cell development, immune regulation and antigen presenting, which probably contribute to the development of ABC-related treatment strategies in various pathogenic diseases.

## Methods

### Single-cell preprocessing, integration and clustering

Four public paired scRNA-seq and scBCR-seq datasets were downloaded from GEO database under accession codes including GSE136035 (SLE) (Gao et al., 2022), GSE307261 (melanoma) (Çakan et al., 2025), GSE315362 (HIV) (Muir et al., 2026), GSE175524 (influenza vaccination) (Wang et al., 2023). For each scRNA-seq dataset, B cells were analyzed by Seurat v5 (Hao et al., 2024) workflow. For SLE and HIV data, we processed the raw gene expression matrix and low-quality cells were dropped from the dataset (more than 200 features and less than 6,000 features, mitochondrial gene ratio<30%). In each study, scRNA-seq and scBCR-seq data were combined according to the corresponding barcodes. B cells with one heavy chain and one light chain were remained. Data normalization, PCA (Principal Component Analysis), UMAP (Uniform Manifold Approximation and Projection) and clustering analysis using the Louvain algorithm with resolution of 1.5 or 2.5 were performed according to the Seurat v5 single-cell integration pipeline. Harmony (Korsunsky et al., 2019) algorithm was used for removing batch effects between diverse samples. We further manually annotated each cell cluster by canonical marker gene expression levels.

### Single-cell data analysis

For scRNA-seq data, we performed differential gene expression analysis, Gene Ontology (GO) analysis, pseudotime analysis, and pathway scoring analysis. We identified the top 50 or 100 upregulated genes (adjusted *P* < 0.05 and log_2_ fold change >1 or >0.585) for each ABC cluster compared with other memory B cell clusters using FindMarkers or FindAllMarkers function from the Seurat package. These genes were subsequently used for pathway enrichment analysis with the enrichGO function in the R package clusterProfiler (PvalueCutoff = 0.05, qvalueCutoff = 0.05) (Wu et al., 2021). We performed pseudotime analysis of Naïve B and memory B cell clusters using R package Monocle3 (Cao et al., 2019).

High expression of *IGHD* and *TCL1A* is characteristic of naïve B cells and marks early B-cell differentiation (Weinstock et al., 2023; Yang et al., 2024). In addition, *MME* (*CD10*) is expressed on early-stage B cells, including transitional and naïve B subsets (Suryani et al., 2010). Guided by these markers, we identified Naive B-PLD4 (*MME* ^+^ *NEIL1* ^+^) as the root for SLE study and Naive B-PLD4-MME-MZB1-NEIL1 cluster as the root for other studies.

Here, we removed batch effects between diverse samples using align_cds function from the Monocle3 package with default parameters. Additionally, we downloaded four signature gene sets from the MSigDB database (https://www.gsea-msigdb.org/gsea/msigdb) under the following terms: “KEGG_B_CELL_RECEPTOR_SIGNALING_PATHWAY”, “GOBP_B_CELL_DIFFERENTIATION”, “GOBP_B_CELL_ACTIVATION_INVOLVED_IN_IMMUNE_RESPONSE”, and “BENNETT_SYSTEMIC_LUPUS_ERYTHEMATOSUS”. The AddModuleScore function from the Seurat package was applied with default parameters to score each pathway in each B cell, and the mean score of all cells within each B cell cluster was then calculated.

### BCR embedding and clustering analysis

AntiBERTy is an antibody-specific bidirectional transformer language model pre-trained on 558 million natural antibody sequences from the Observed Antibody Space (OAS) (Devlin et al.; Ruffolo et al., 2021). Embeddings from AntiBERTy act as a contextual representation, which could cluster BCRs with similar biochemical properties and structural features in the embedding space. Ruffolo et al. have proved that AntiBERTy has learned some distinguishing structural features of CDR loops through sequence pre-training alone, validated on 3,467 paired antibody sequences with experimentally determined structures in the PDB (Ruffolo et al., 2023). While exact CDR3 identity was used to define strict clonal lineages, embedding-based clustering could capture broader patterns of sequence similarity that may reflect convergent BCR responses to shared antigenic stimuli. For each scBCR-seq dataset, we performed BCR CDR3 embedding analysis using AntiBERTy. Each BCR CDR3 sequence was encoded as a 512-dimensional numeric vector. Namely, similar CDR3 sequence would obtain correlated CDR3 embeddings. For each B cell, CDR3 embeddings of heavy and light chain were joined in order to represent the embedding of the whole CDR3 sequence. We performed PCA and selected the top 50 principal components (PCs) to calculate cosine distance matrix between diverse BCR CDR3s. Previous studies have typically clustered BCR CDR3 sequences using a normalized Levenshtein distance of 0.15 or 0.1 (Yang et al., 2025). The normalized Levenshtein distance between two CDR3 sequences was defined as the ratio of their Levenshtein distance to the maximum sequence length. For paired heavy-chain and light-chain CDR3s, the overall distance was calculated as the average of the normalized Levenshtein distances of the respective chains. For each dataset, we decided the optimal embedding-based distance cutoff by performing gradient analysis of pairwise normalized Levenshtein distances of CDR3 sequences across a range of clustering cutoffs from 0.04 to 0.11, yielding optimal cutoffs of 0.08 for SLE, 0.06 for melanoma, 0.10 for HIV, and 0.06 for influenza vaccination. For each sample or donor, we constructed a CDR3-based graph by linking CDR3 pairs with a cosine distance below the optimal cutoff, and then performed CDR3 clustering on this graph, yielding CDR3-based BCR networks.

### Calculation of clonally expanded BCR transcriptomic diversity

We defined clonally expanded BCRs as those composed of at least 2 B cells sharing identical CDR3 sequences. For each clonally expanded BCR, we quantified its transcriptomic diversity by calculating the average pairwise cosine distance of its constituent B cells in the PCA space of gene expression profiles using top 50 PCs.

### Calculation of BCR diversity and BCR connectivity in each sample

Given that BCR somatic hypermutation drives the continuous evolution of BCRs and creates a family of similar BCRs with diverse antigen-targeting efficiencies (Zhang et al., 2022), we evaluated the BCR connectivity based on our BCR clustering results, which accounted for both CDR3 mutations and structural similarity rather than relying solely on exact CDR3 matches. BCR diversity was calculated as the Shannon entropy of the BCR clusters. We defined BCR connectivity as the proportion of cells that co-clustered with at least one other cell. Specifically, for each sample, cells whose BCR sequences were grouped into clusters containing ≥2 cells were considered “connected” and BCR connectivity was calculated as the number of such cells divided by the total number of cells in that sample. Furthermore, for each cell cluster, we also calculated the BCR diversity and BCR connectivity among its constituent B cells.

### BCR similarity analysis across diverse cell clusters

For each study, we performed pairwise comparisons of BCRs across diverse cell clusters. For each pair of cell clusters within a sample, we defined a BCR similarity/connectivity score as the proportion of cells whose BCRs were co-clustered across the two clusters. Specifically, a cell from cluster A was defined as connected with cluster B when its BCR was co-clustered with BCRs from cluster B. The similarity/connectivity score for a cluster pair was then computed as the ratio of the number of connected cells to the total number of cells in the two cell clusters. This yielded a score for each cluster pair in each sample. These scores were then averaged across samples and phenotypic conditions for visualization. The BCR connectivity network was visualized by heatmaps or chord diagrams using R package ‘circlize’ (Gu et al., 2014).

### BCR similarity analysis across diverse time points

For the melanoma and influenza vaccination dataset, we performed embedding-based BCR clustering analysis on all BCRs for each donor using the optimal clustering cutoff as described in “**BCR embedding and clustering analysis”**. Using Sankey diagrams, we visualized the BCR connectivity of B cell clusters across time points for each donor. For each time point and BCR cluster in each donor, we selected the most dominant cell cluster, focusing on BCRs shared across multiple time points for visualization. For HIV dataset, the analysis was performed for each replicate group.

### Correlation of B cell transcriptomic with BCR embedding distance

To explore the link between B cell transcriptional states and BCR sequence features, we performed correlation analysis of intra-cluster gene expression distance and CDR3 embedding distance across a range of clustering cutoffs. Under these settings, relatively inclusive clustering cutoffs yielded a sufficient number of BCR clusters containing at least two cells per cluster, a prerequisite for computing pairwise distances across clusters. For each BCR cluster comprising at least two cells in a given sample, the average gene expression distance and average CDR3 embedding distance across all pairwise cells were calculated. Gene expression distance was defined as the cosine distance in the PCA space based on the top 50 PCs. Similarly, BCR embedding distance was defined as the cosine distance in the embedding space using the top 50 PCs. Correlation analysis was subsequently performed at the BCR cluster level to evaluate the association between B cell transcriptomic diversity and corresponding BCR diversity.

### Comparative analysis of pre- versus post-vaccination BCRs for influenza patients

To quantify BCR repertoire dynamics in response to influenza vaccination, we employed two strategies to evaluate pre- versus post-vaccination BCR similarity. Correspondingly, we calculated the cosine distance matrix based on the top 50 principal components (PCs) of the BCR CDR3 embeddings, with diagonal entries set to positive infinity for each patient.

We first constructed a nearest-neighbor graph to cluster similar BCRs and calculated the proportion of BCR clusters containing both pre- and post-vaccination BCRs per patient. For those clusters containing BCRs from both time points, we further calculated the purity of BCRs originating from the two time points.

Additionally, we constructed two similarity matrices based on BCR cosine distance and ranking. The distance-based similarity matrix was defined using the cosine similarity of BCR CDR3 embeddings. The ranking-based similarity matrix was calculated by assigning edge weights inversely proportional to neighbor rank according to cosine distance: the nearest neighbor received a weight of 1, the second nearest 1/2, the third nearest 1/3, and so forth. For each pre-vaccination BCR within a given patient, we computed the average scores from the two similarity matrices. The same procedure was applied to compute similarity scores for post-vaccination CDR3s.

### Integrative analysis of ABCs

To harmonize ABCs from different studies and donors, we performed data integration using reciprocal PCA (RPCA) as implemented in the Seurat v5 workflow. ABC clustering was initially performed at a resolution of 1.0, and extremely similar cell clusters were manually merged based on both canonical marker gene signatures and pairwise correlation of cluster-specific upregulated genes, ultimately yielding nine distinct cell clusters.

Batch effect correction performance was evaluated by computing the local Inverse Simpson’s Index (LISI) for each cell at both the study and donor levels based on the integrated RPCA space, using the lisi R package (Korsunsky et al., 2019). To assess the generalizability of the obtained clusters, we performed 100 independent iterations of cell-level random 80/20 train-test splits and cell type label transfer analysis as implemented in Seurat v5. In each iteration, anchors were identified between training and testing sets using Seurat’s FindTransferAnchors function, and the testing set labels were predicted using Seurat’s TransferData function. Accuracies and F1 scores were calculated to evaluate the clustering performance. We further performed leave-one-dataset-out cross-validation, in which cells from each study were iteratively held out as the testing set while the remaining datasets served as the training reference. Cluster labels for the held-out cells were predicted via label transfer, and prediction accuracies were computed accordingly. This procedure was repeated for each of the four datasets (SLE, melanoma, HIV, and influenza vaccination).

To assess the phenotype preference of the resulting ABC clusters, we calculated the ratio of observed to expected cell numbers (Ro/e) for each ABC cluster across different phenotypes. The expected cell numbers for each cluster in a given tissue were derived from the chi-square test, where an Ro/e value greater than one indicates enrichment of a cluster in a specific phenotype (Zhang et al., 2018). Given that each ABC cluster showed distinct patterns of phenotype-specific enrichment or depletion, we performed BCR similarity analysis at the cluster or phenotype level (pooling cells from all related samples). Specifically, we performed embedding-based BCR similarity analysis between all pairs of cell clusters or phenotypic groups across all related ABCs, using a uniform cosine distance cutoff of 0.1 determined by gradient analysis. This approach aggregated information across samples and donors to reveal conserved BCR connectivity patterns between ABC subpopulations, rather than assessing connectivity at the individual sample level. For two given groups A and B, a cell was defined as connected if its BCR co-clustered with BCRs from the other group. The BCR connectivity score of group A with group B was computed as the number of connected cells in A with B divided by the total number of cells in A. Similarly, the connectivity score of group B with group A was calculated as the number of connected cells in B with A divided by the total number of cells in B. To evaluate BCR connectivity between phenotypes while excluding donor-specific effects, we performed within-donor permutation tests. Specifically, for each donor, phenotype labels were randomly shuffled 1,000 times, and the BCR connectivity score was recalculated for each permutation. The empirical *P* value was defined as the fraction of permuted connectivity scores that exceeded the observed score.

## Data Availability

All public datasets used in this study were described in the Methods section. The public scRNA-seq and scBCR-seq datasets can be downloaded from GEO database under accession codes including GSE136035, GSE307261, GSE315362 and GSE175524. The codes and corresponding data in this manuscript were deposited at https://doi.org/10.5281/zenodo.21070889.

## Supplementary material

SUPPLEMENTARY TABLE S1 Cell count of each cell cluster across all samples for each scRNA-seq dataset.

SUPPLEMENTARY TABLE S2 Cell count of each ABC cluster across all scRNA-seq samples for 23,373 ABCs, and differentially expressed genes identified for each ABC cluster.
