# Supplementary material for: Single-cell multi-omics reveals circulating B cell heterogeneity across diverse disease contexts
Source: Front Cell Dev Biol. 2026 Aug 4;14:1897676. doi: 10.3389/fcell.2026.1897676 (PMC13481364; doi:10.3389/fcell.2026.1897676)
Supplement: Supplementary file 1 [file DataSheet1.pdf]

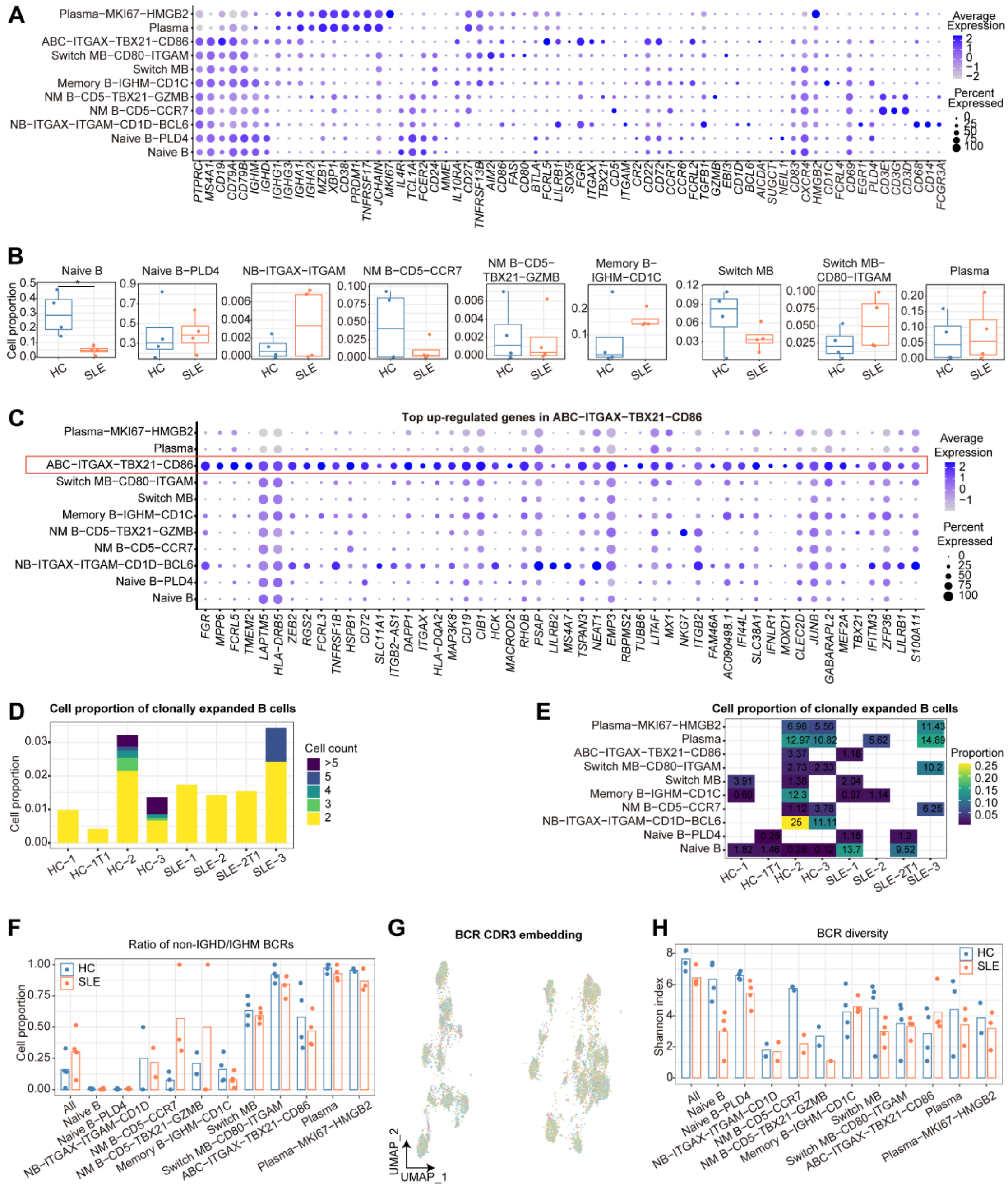

**Figure S1. Strong ABC activation and BCR signaling in SLE patients.**
(**A**) Dot plot showing the expression of canonical marker genes in each cell cluster. Point size indicates the percentage of cells expressing the gene in the cell cluster. Point color indicates the scaled gene expression distribution across diverse phenotypes. (**B**) Comparisons of cell cluster proportions between diverse phenotypes. (**C**) Dot plot showing the expression of top 50 up-regulated genes in ABCs. (**D**) Distribution of diverse clonal levels for clonally expanded B cells in each sample. (**E**) Proportion of clonally expanded B cells in each cell cluster and sample, with numbers indicating the percentage of clonally expanded B cells. (**F**) Cell proportion of BCRs lacking IgD or IgM isotype usage in all B cells and across diverse cell clusters and phenotypes. (**G**) UMAP plot of BCRs based on CDR3 embeddings, colored by cell cluster. (**H**)

Distribution of average BCR diversity in all B cells and diverse cell clusters across different phenotypes with a BCR clustering cutoff of 0.08.

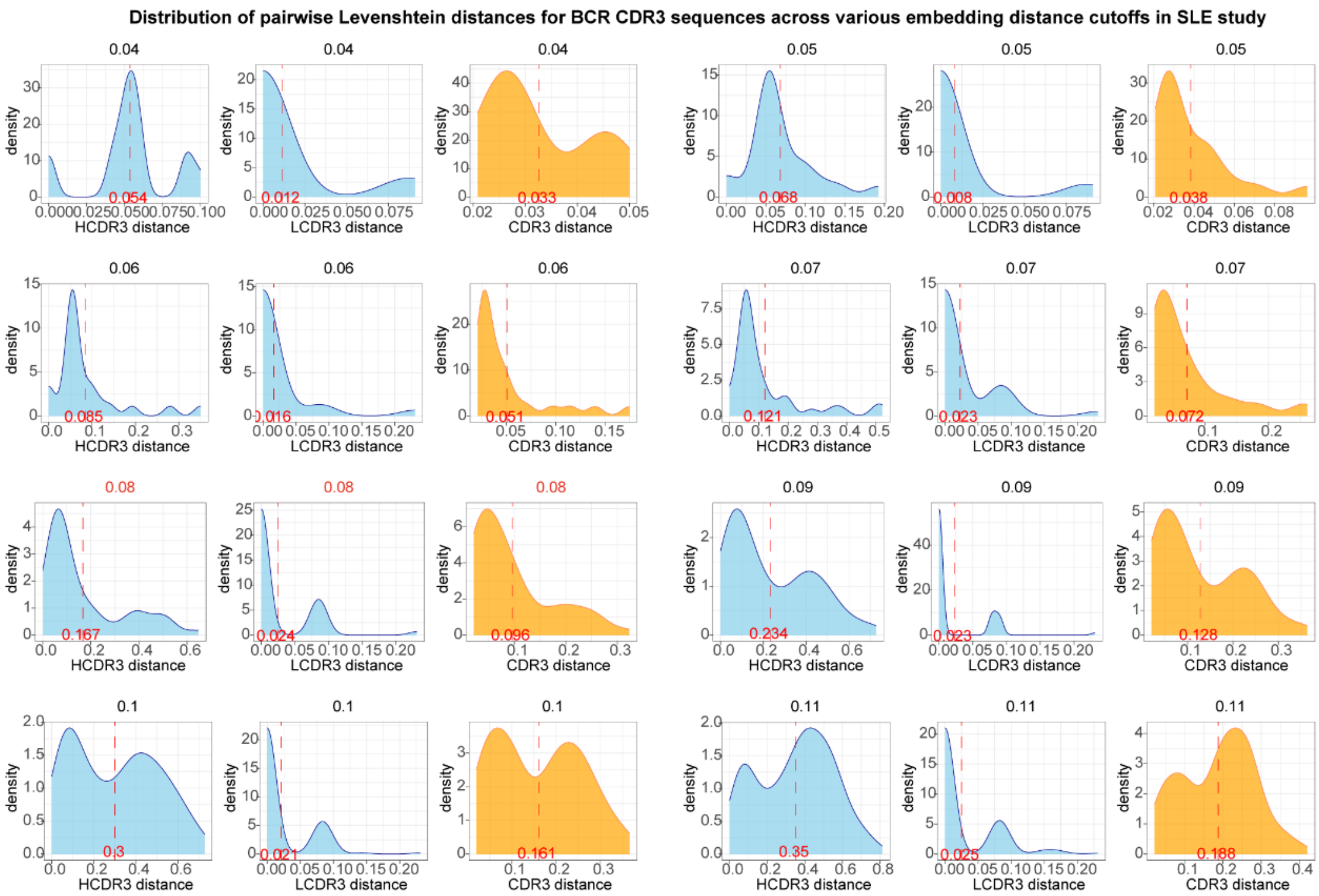


**Figure S2. Distribution of pairwise Levenshtein distances among BCR CDR3 sequences across various embedding distance cutoffs in the SLE study.**

Density plots showing the distribution of pairwise Levenshtein distances among all BCR CDR3 sequences (heavy chain, light chain and paired) at varying embedding distance cutoffs in the SLE study. Each subplot title indicates the corresponding embedding distance cutoff, while the red dashed line marks the mean Levenshtein distance, with its value annotated adjacent to the line.

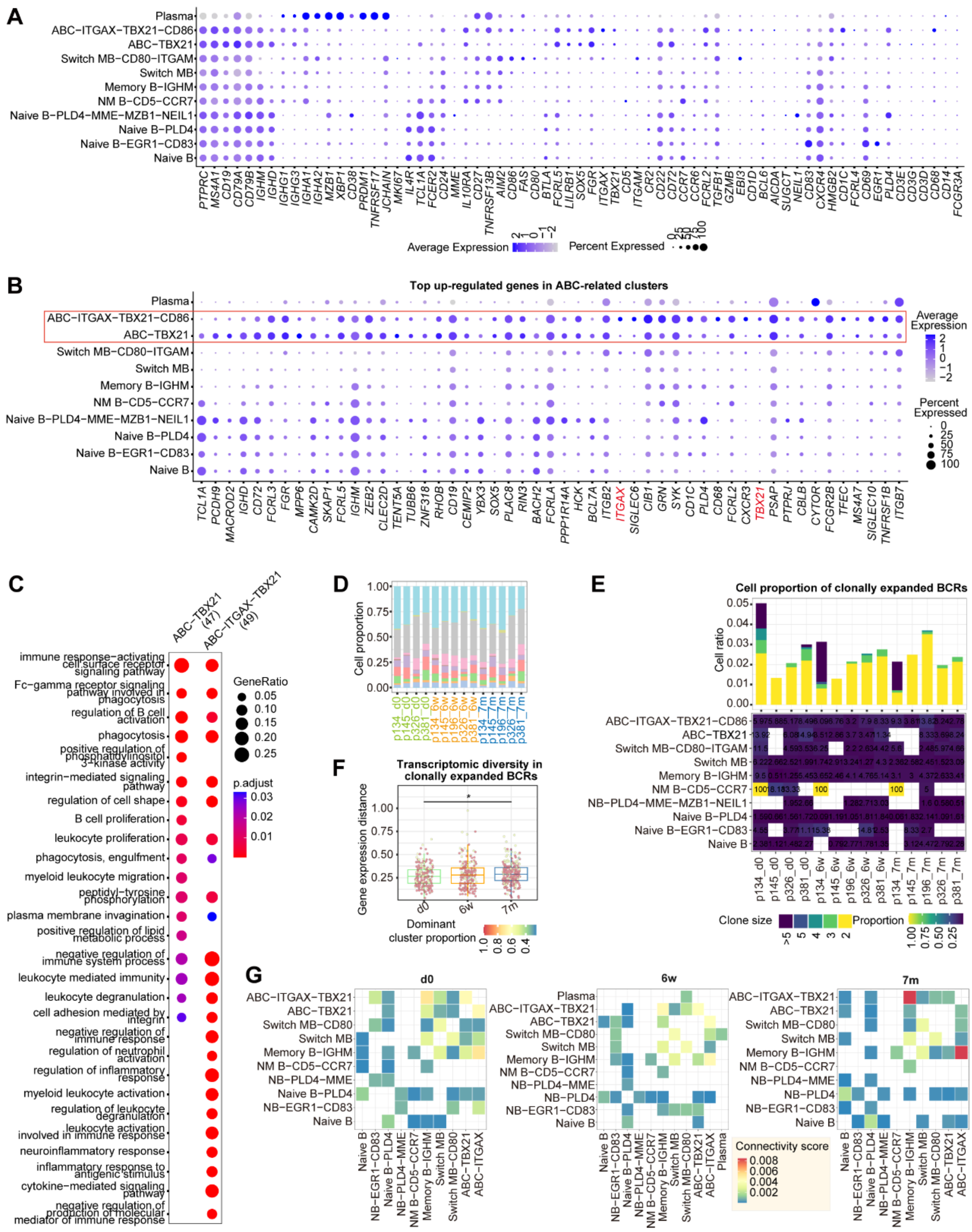

**Figure S3. Dynamics of B cells in melanoma patients during immunotherapy.**
(**A**) Dot plot showing the expression of canonical marker genes in each cell cluster. Point size indicates the percentage of cells expressing the gene in the cell cluster. Point color indicates the scaled gene expression distribution across diverse phenotypes. (**B**) Dot plot showing the expression of top significant up-regulated genes in ABC clusters. (**C**) Top enriched pathways using top 50 up-regulated genes in each ABC cluster. Point size indicates the ratio of genes involved with the pathway to the total number of genes in the cluster. Point color indicates the adjusted *P* value calculated using enrichGO function. (**D**) Distribution of various cell clusters in each sample. (**E**) The top panel indicates the distribution of diverse clonal levels for clonally expanded B cells in each

sample. The bottom panel indicates the proportion of clonally expanded B cells in each cell cluster and sample, with numbers indicating the percentage of clonally expanded B cells. (**F**) Comparisons of transcriptomic diversity for those clonally expanded BCRs between diverse time points. Each point represents a B cell clone composed of B cells with identical BCR CDR3 sequences. Point color represents the dominant cell cluster proportion in the clone. Statistical significance was assessed using Wilcoxon rank-sum test. (**G**) Heatmaps showing the average connectivity score of BCRs between various cell clusters at each time point with a BCR clustering cutoff of 0.06.

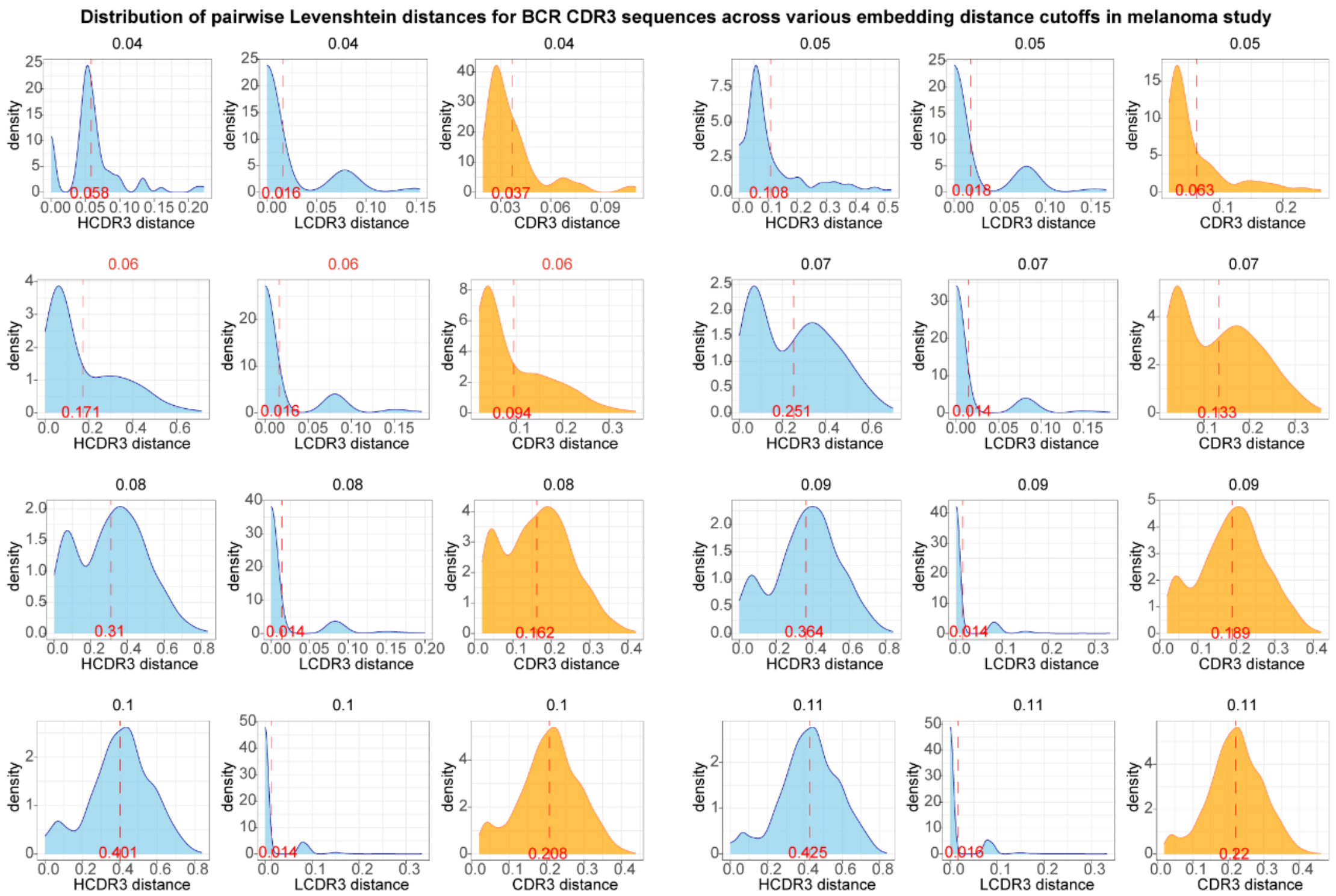


**Figure S4. Distribution of pairwise Levenshtein distances among BCR CDR3 sequences across various embedding distance cutoffs in the melanoma study.**

Density plots showing the distribution of pairwise Levenshtein distances among all BCR CDR3 sequences (heavy chain, light chain and paired) at varying embedding distance cutoffs in the melanoma study. Each subplot title indicates the corresponding embedding distance cutoff, while the red dashed line marks the mean Levenshtein distance, with its value annotated adjacent to the line.

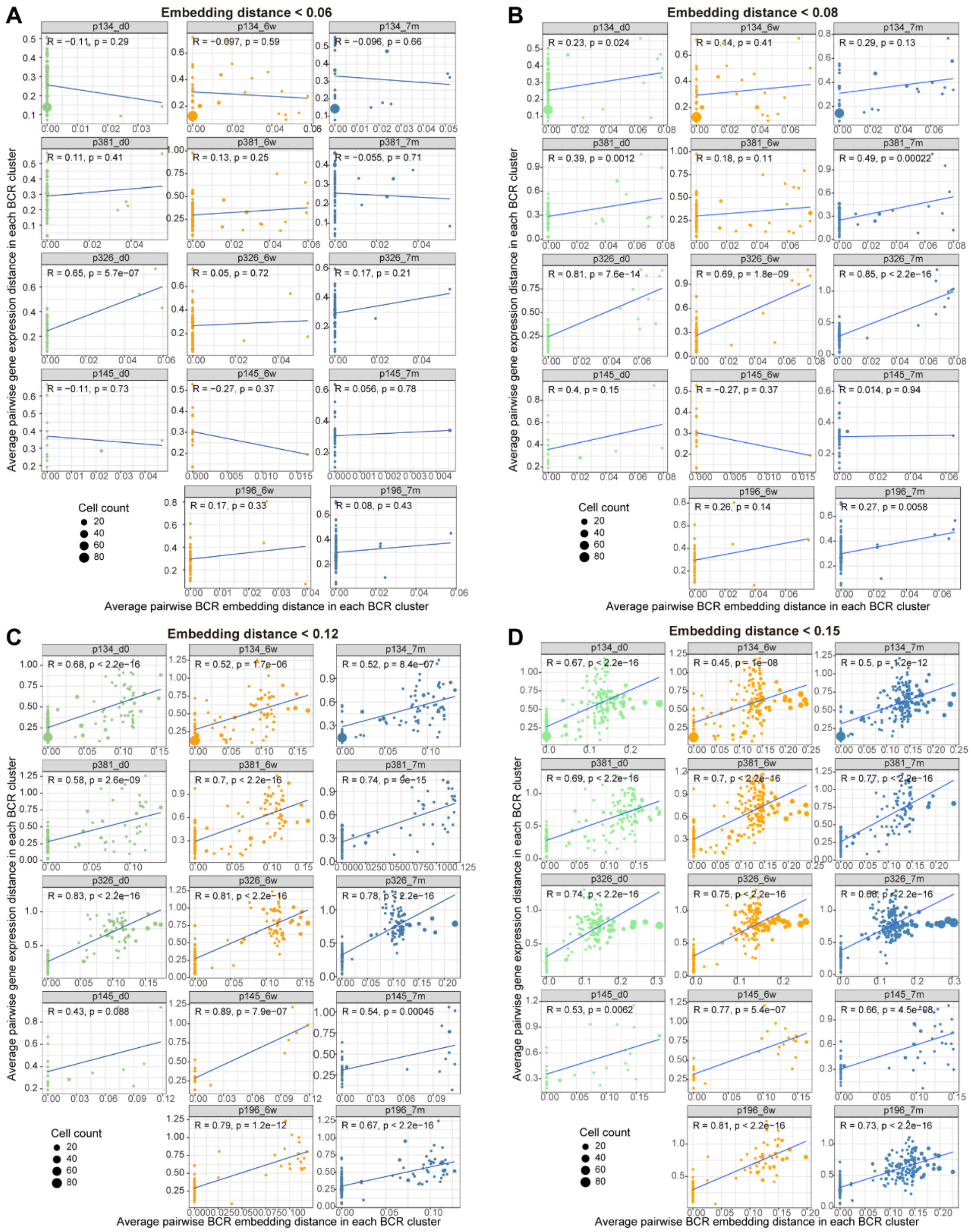


**Figure S5. Correlation between BCR embedding distance with gene expression distance in the melanoma study.**

(**A-D**) Pearson's correlation between BCR diversity (average pairwise BCR CDR3 embedding distance) with transcriptomic diversity (average pairwise gene expression distance) across all BCR clusters (at least 2 cells) in each sample using various clustering cutoffs of 0.06, 0.08, 0.12 and 0.15. Each point represents a BCR cluster. Point size indicates the number of cells in the BCR cluster. Pearson's correlation coefficient and *P* value are indicated on the top of each panel.

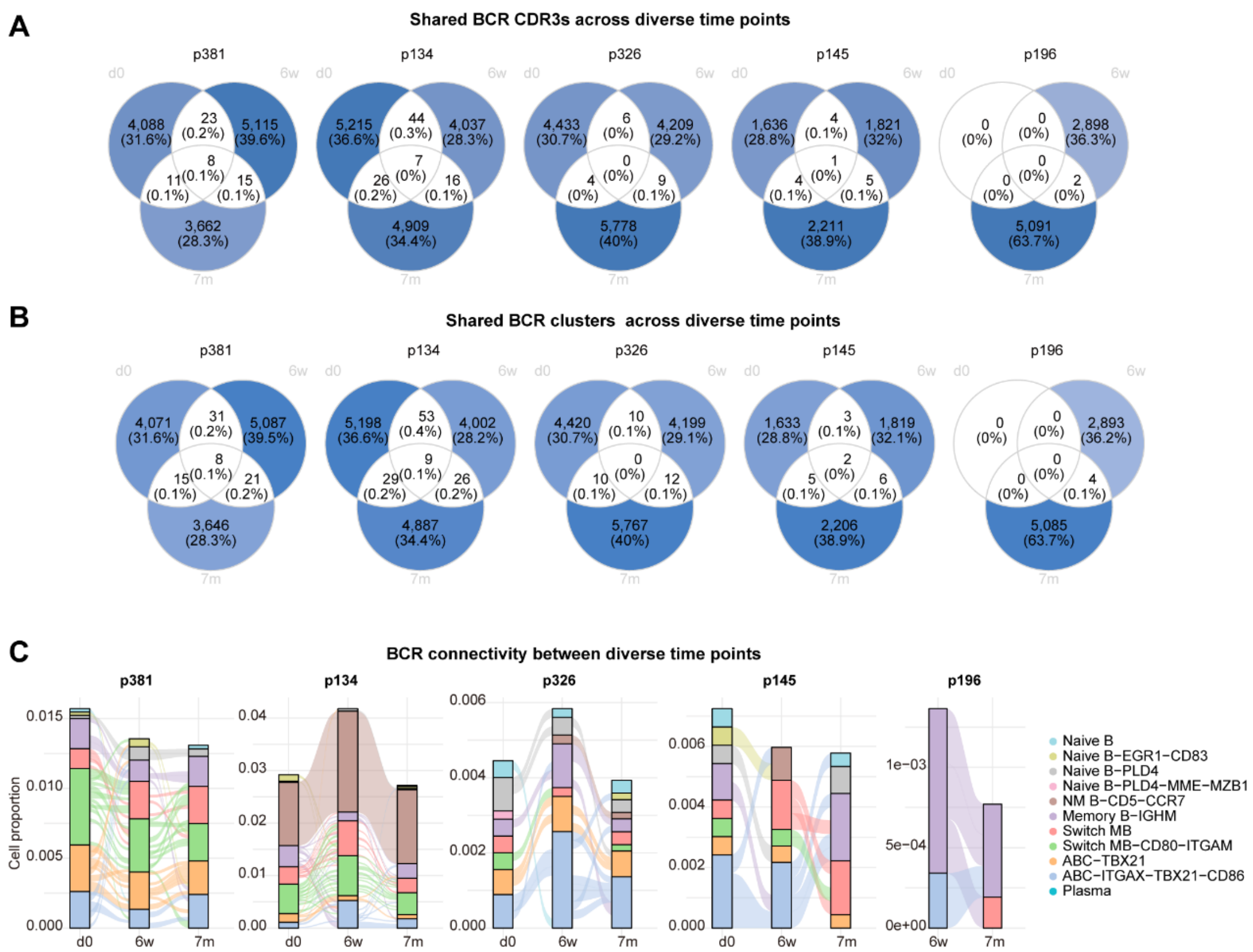


**Figure S6. Dynamics of B cells in melanoma patients during immunotherapy.** (**A**) Venn diagrams showing the intersections of BCR CDR3s across different time points for each patient. Overlapping BCR CDR3 numbers and proportions are indicated in each region. (**B**) Venn diagrams showing the intersections of BCR clusters across different time points for each patient with a BCR clustering cutoff of 0.06. Overlapping BCR cluster numbers and proportions are indicated in each region. (**C**) Sankey plots showing the cell cluster distribution of connected BCRs among different time points. Ribbons indicate that cells from two different time points are clustered together within the same BCR cluster. Ribbon color indicates B cell cluster. The y-axis represents the aggregated connected cell proportions of all dominant cell clusters at each time point in each BCR cluster.

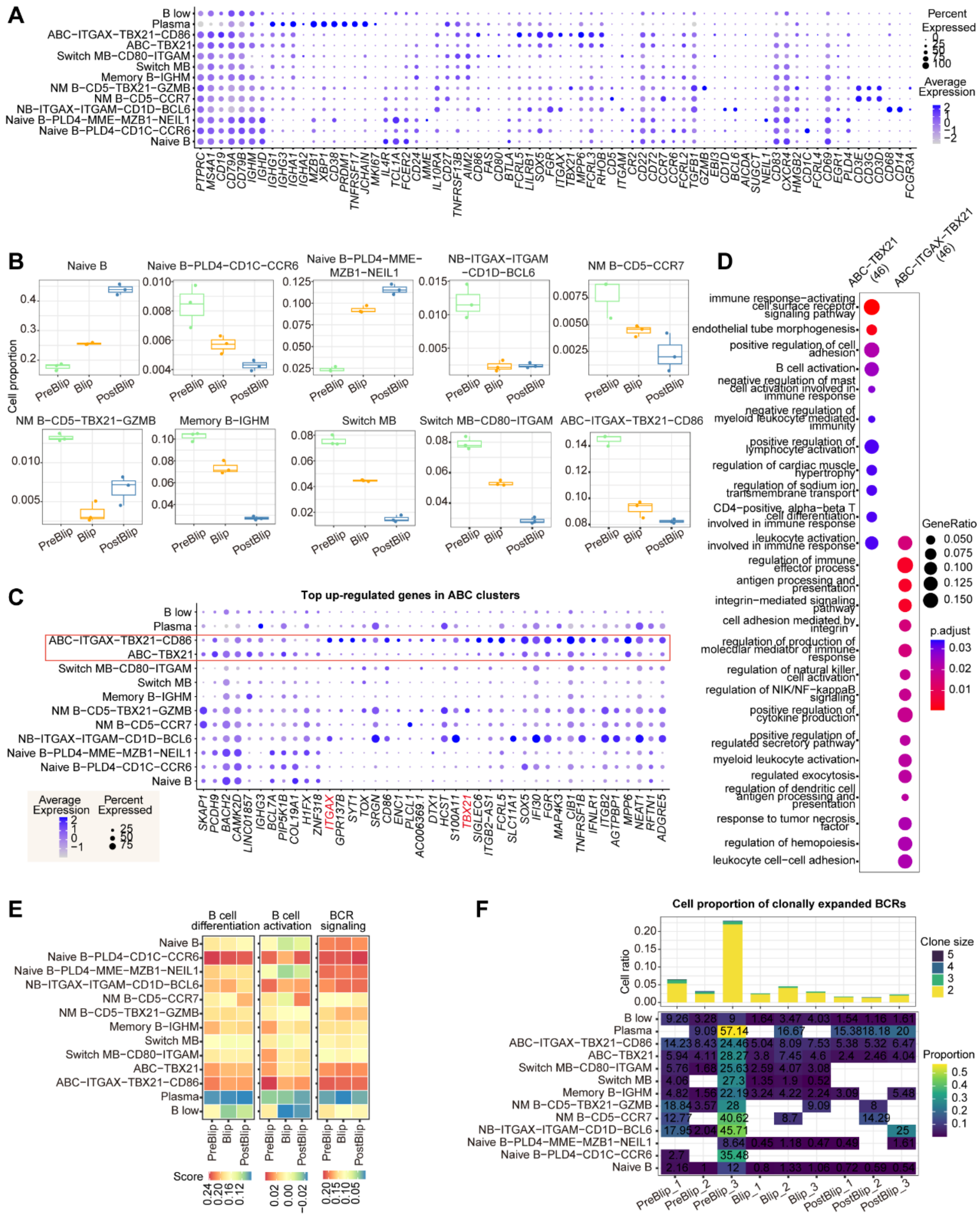

**Figure S7. Dynamics of B cell response to HIV viral blip in one elite controller.** (**A**) Dot plot showing the expression of canonical marker genes in each cell cluster. (**B**) Comparisons of cell cluster proportions between various time points. (**C**) Dot plot showing the expression of top significant up-regulated genes in ABC clusters. (**D**) Top enriched pathways using top 50 up-regulated genes in each ABC cluster. (**E**) Distribution of average signature scores for different pathways across diverse cell clusters and time points. (**F**) The top panel indicates the clonal expansion levels in each sample, with cells categorized by the clone size of their corresponding clones. The bottom panel indicates the proportion of clonally expanded B cells in each cell cluster and sample, with numbers indicating the percentage of clonally expanded B cells.

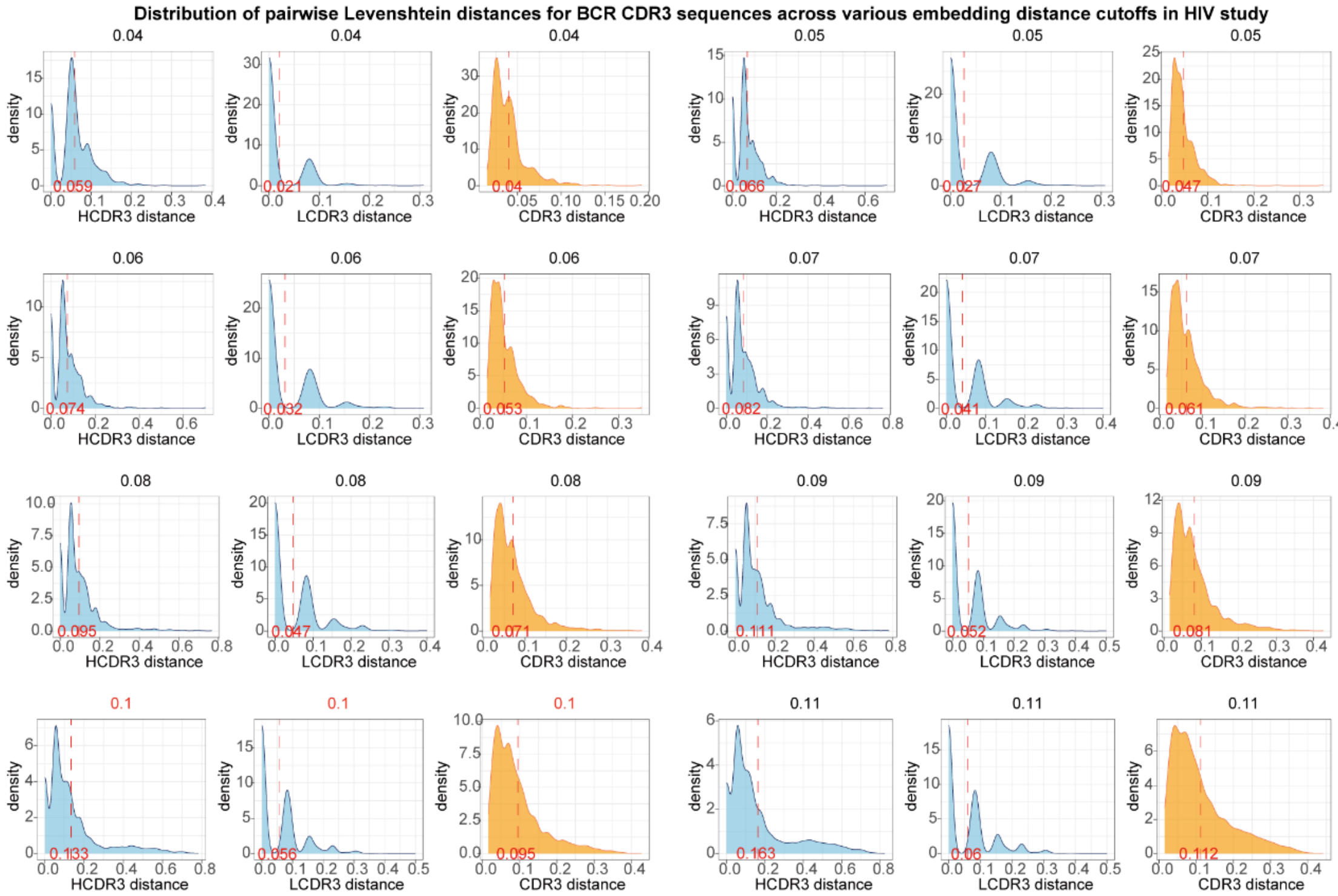


**Figure S8. Distribution of pairwise Levenshtein distances among BCR CDR3 sequences across various embedding distance cutoffs in the HIV study.**

Density plots showing the distribution of pairwise Levenshtein distances among all BCR CDR3 sequences (heavy chain, light chain and paired) at varying embedding distance cutoffs in the HIV study. Each subplot title indicates the corresponding embedding distance cutoff, while the red dashed line marks the mean Levenshtein distance, with its value annotated adjacent to the line.

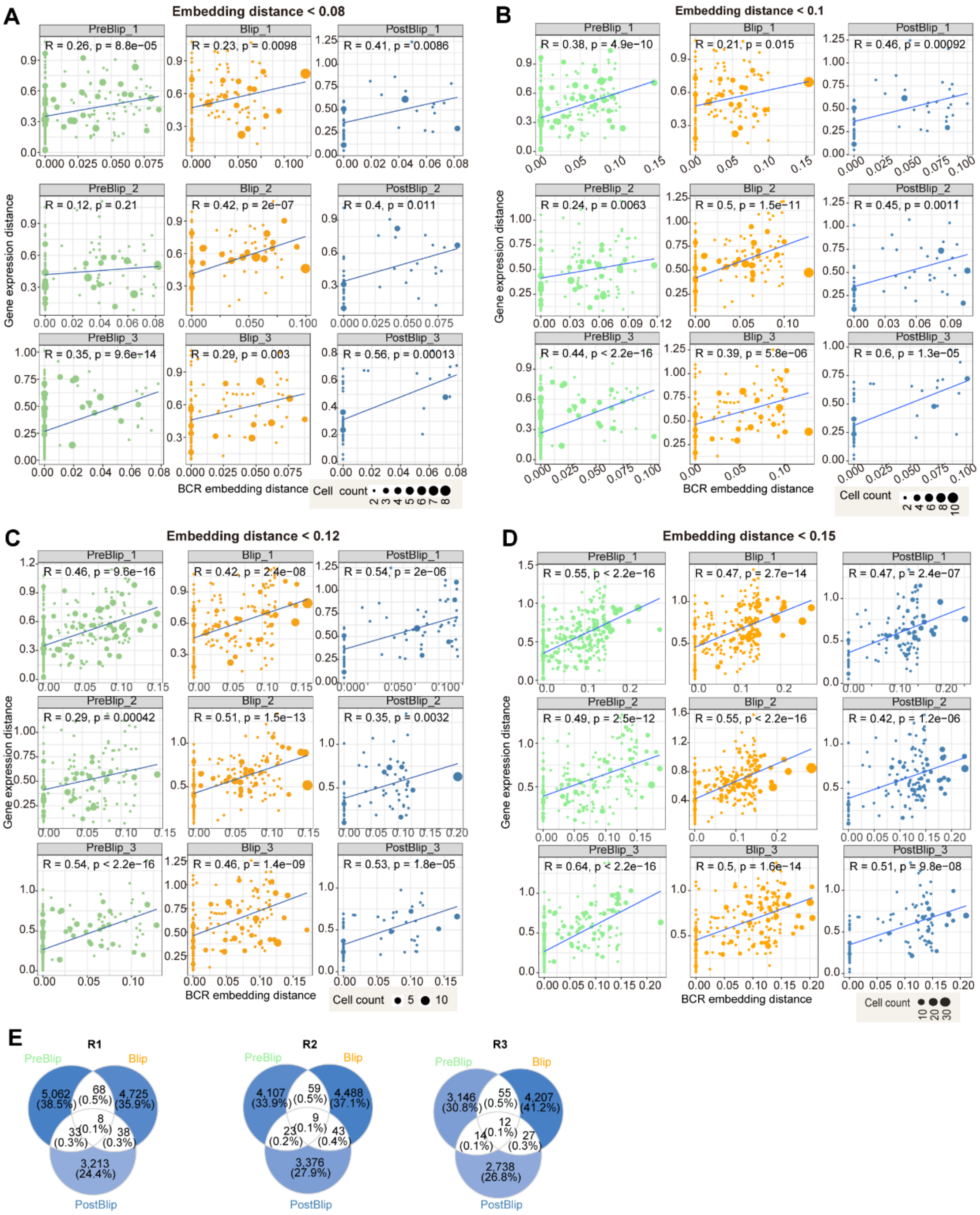


**Figure S9. Dynamics of B cell response to HIV viral blip in one elite controller.** (**A-D**) Pearson's correlation between BCR diversity (averaged BCR CDR3 embedding distance) with transcriptomic diversity (averaged gene expression distance) across all BCR clusters (at least 2 cells) in each sample using various clustering cutoffs of 0.08, 0.1, 0.12 and 0.15. Each point represents a BCR cluster. Point size indicates the number of cells in the BCR cluster. Pearson's correlation coefficient and *P* value are indicated on the top of each panel. (**E**) Venn diagrams showing the intersections of BCR CDR3s across different time points for each patient. Overlapping BCR CDR3 numbers and proportions are indicated in each region.

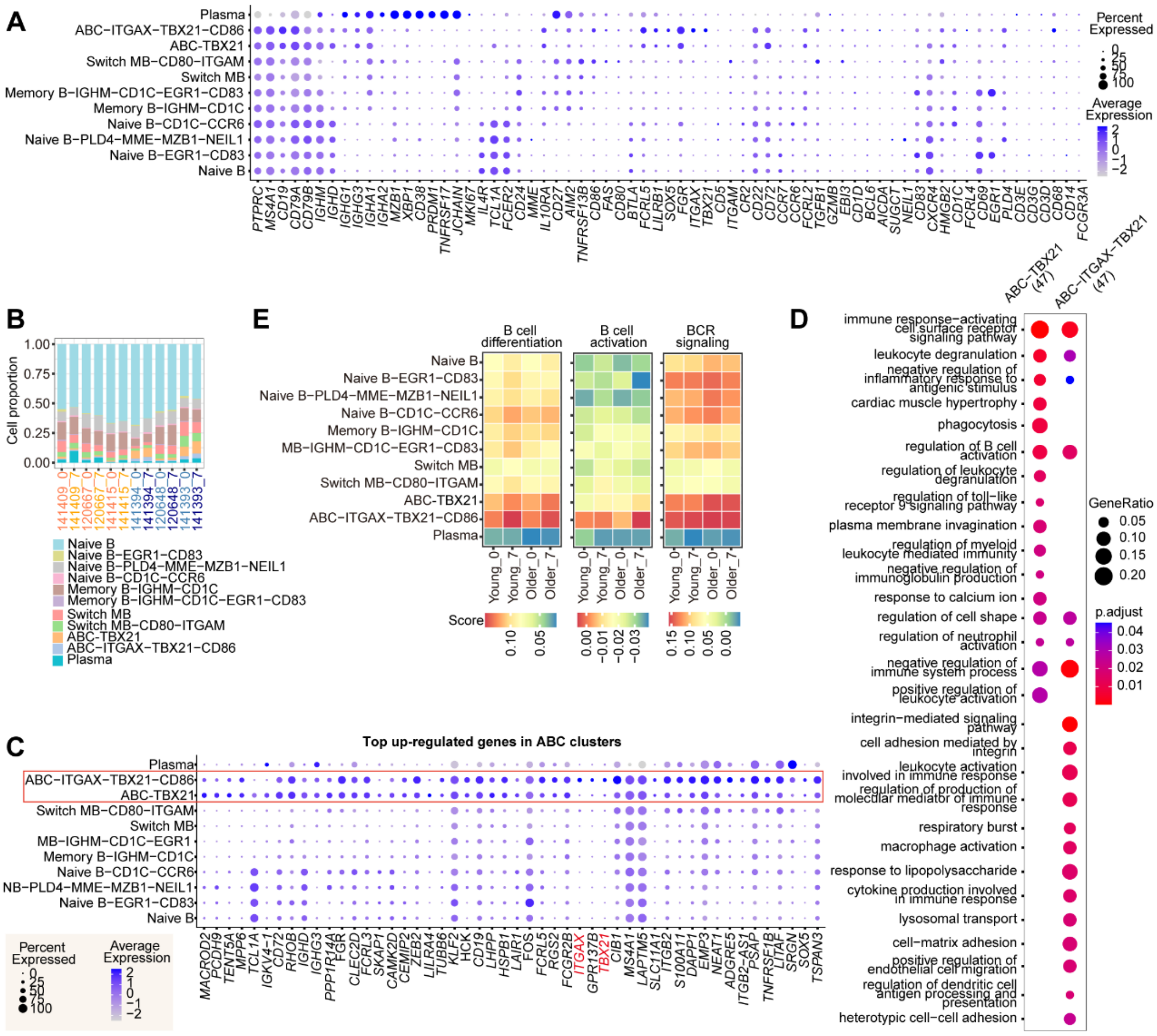

**Figure S10. Distinct B cell responses to influenza vaccination in young and older responders.**

(**A**) Dot plot showing the expression of canonical marker genes in each cell cluster. (**B**) Distribution of cell cluster proportions across samples. (**C**) Dot plot showing the expression of top significant up-regulated genes in ABC clusters. (**D**) Top enriched pathways using top 50 up-regulated genes in each ABC cluster. (**E**) Distribution of average signature scores for different pathways across diverse cell clusters and time points.

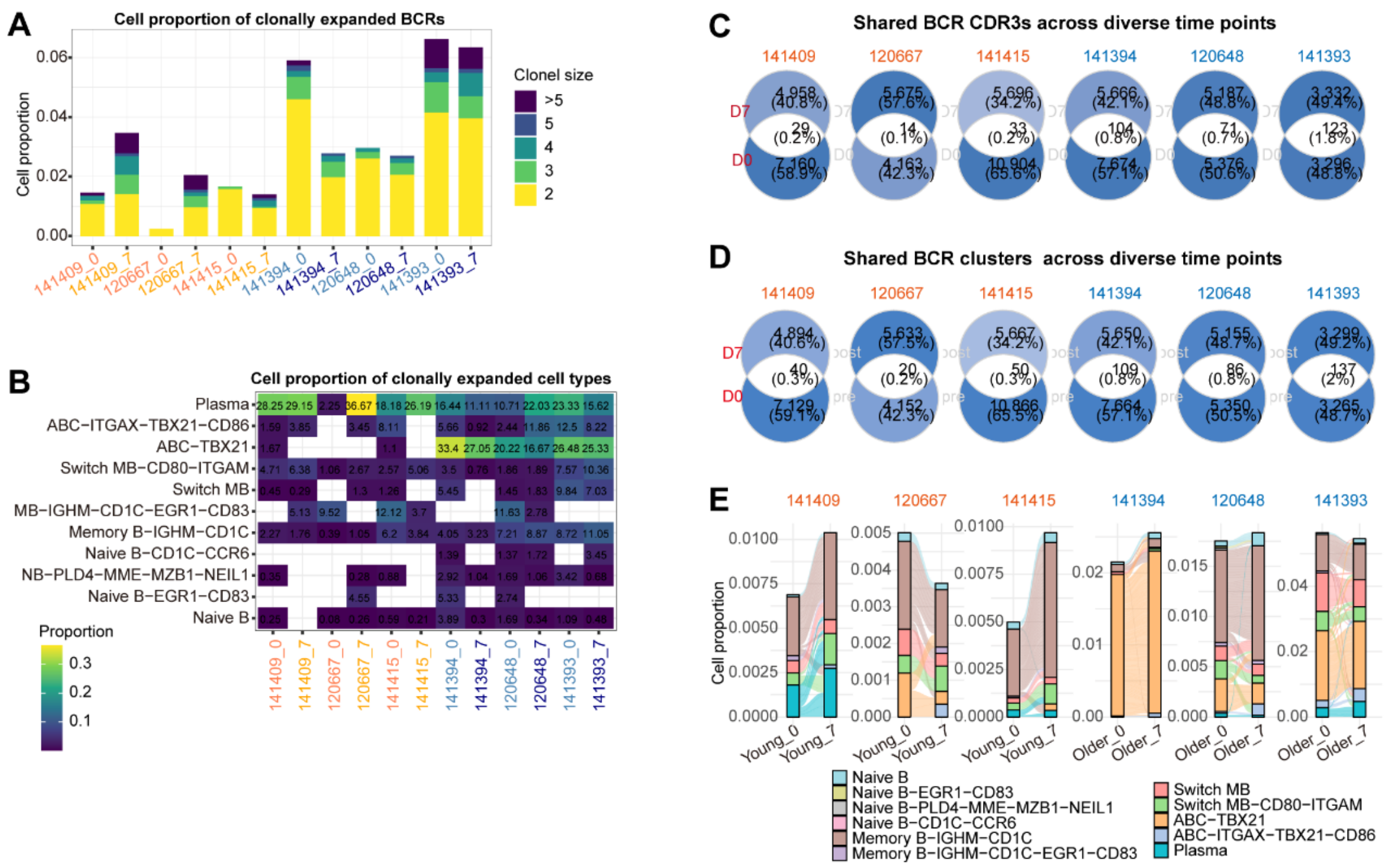

**Figure S11. Distinct B cell responses to influenza vaccination in young and older responders.**

(**A**) Distribution of diverse clonal levels in each sample. (**B**) Proportion of clonally expanded B cells in each cell cluster and sample, with numbers indicating the percentage of clonally expanded B cells. (**C**) Venn diagrams showing the intersections of BCR CDR3s across different time points for each subject. Overlapping BCR CDR3 numbers and proportions are indicated in each region. (**D**) Venn diagrams showing the intersections of BCR clusters across different time points for each subject with a BCR clustering cutoff of 0.06. Overlapping BCR cluster numbers and proportions are indicated in each region. (**E**) Sankey plots showing the cell cluster distribution of connected BCRs among different time points. Ribbons indicate that cells from two different time points are clustered together within the same BCR cluster. Ribbon color indicates B cell cluster. The y-axis represents the aggregated connected cell proportions of all dominant cell clusters at each time point in each BCR cluster.

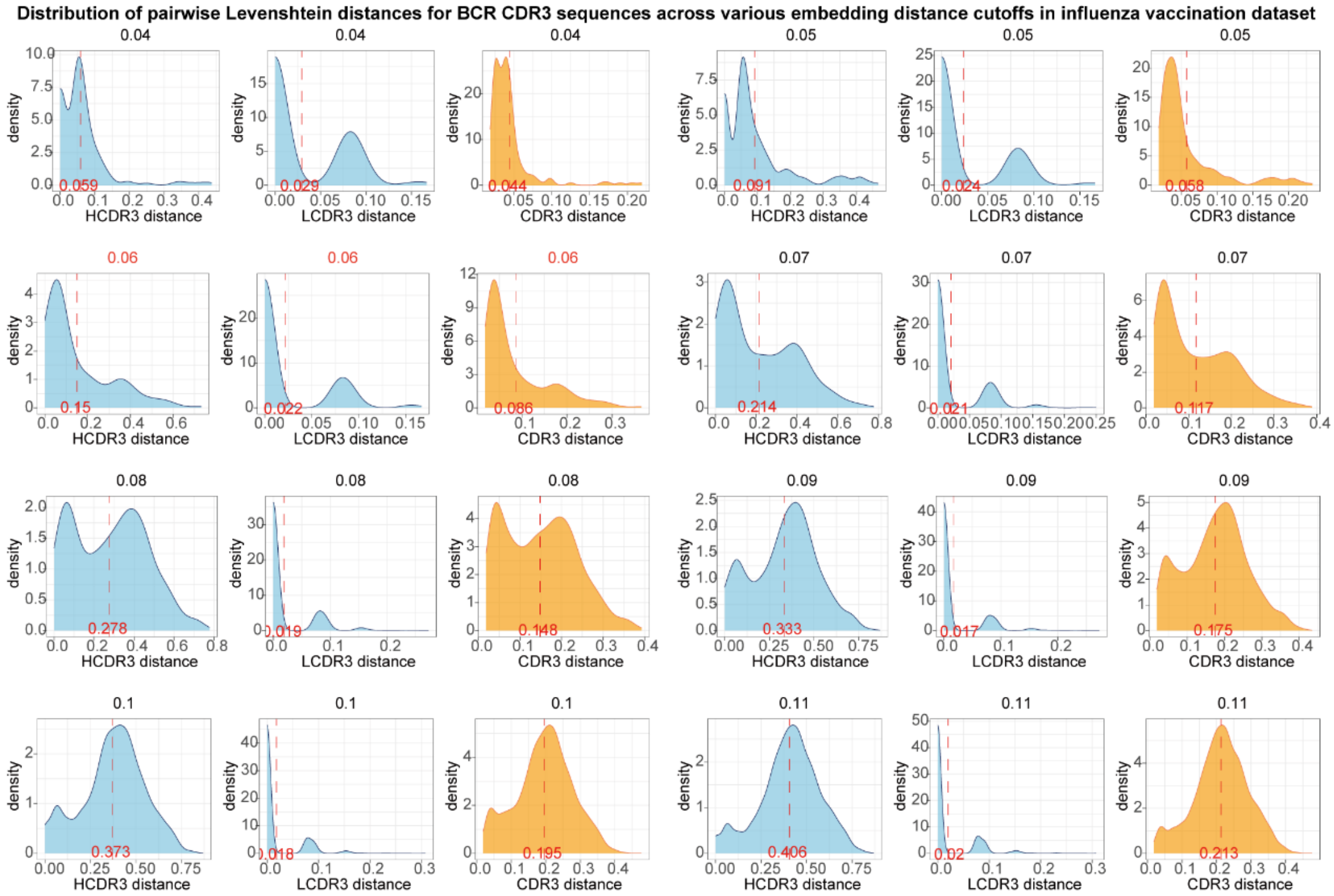


**Figure S12. Distribution of pairwise Levenshtein distances among BCR CDR3 sequences across various embedding distance cutoffs in the influenza vaccination study.**

Density plots showing the distribution of pairwise Levenshtein distances among all BCR CDR3 sequences (heavy chain, light chain and paired) at varying embedding distance cutoffs in the influenza vaccination study. Each subplot title indicates the corresponding embedding distance cutoff, while the red dashed line marks the mean Levenshtein distance, with its value annotated adjacent to the line.

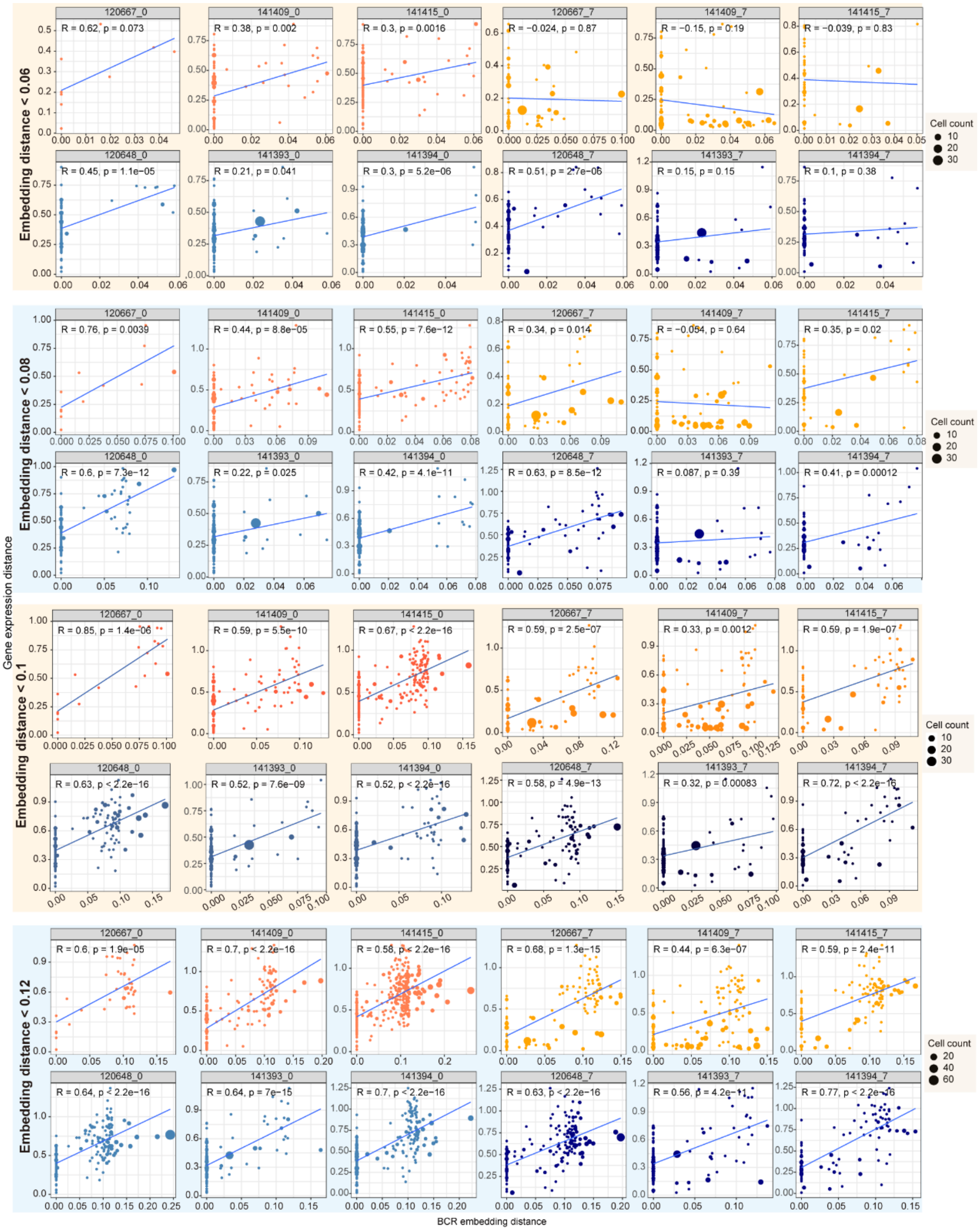


**Figure S13. Correlation between BCR embedding distance with gene expression distance in the influenza vaccination study.**

Pearson's correlation between BCR diversity (average pairwise BCR CDR3 embedding distance) with transcriptomic diversity (average pairwise gene expression distance) across all BCR clusters (at least 2 cells) in each sample using various clustering cutoffs of 0.06, 0.08, 0.1 and 0.12. Each point represents a BCR cluster. Point size indicates the number of cells in the BCR cluster. Pearson's correlation coefficient and *P* value are indicated on the top of each panel.

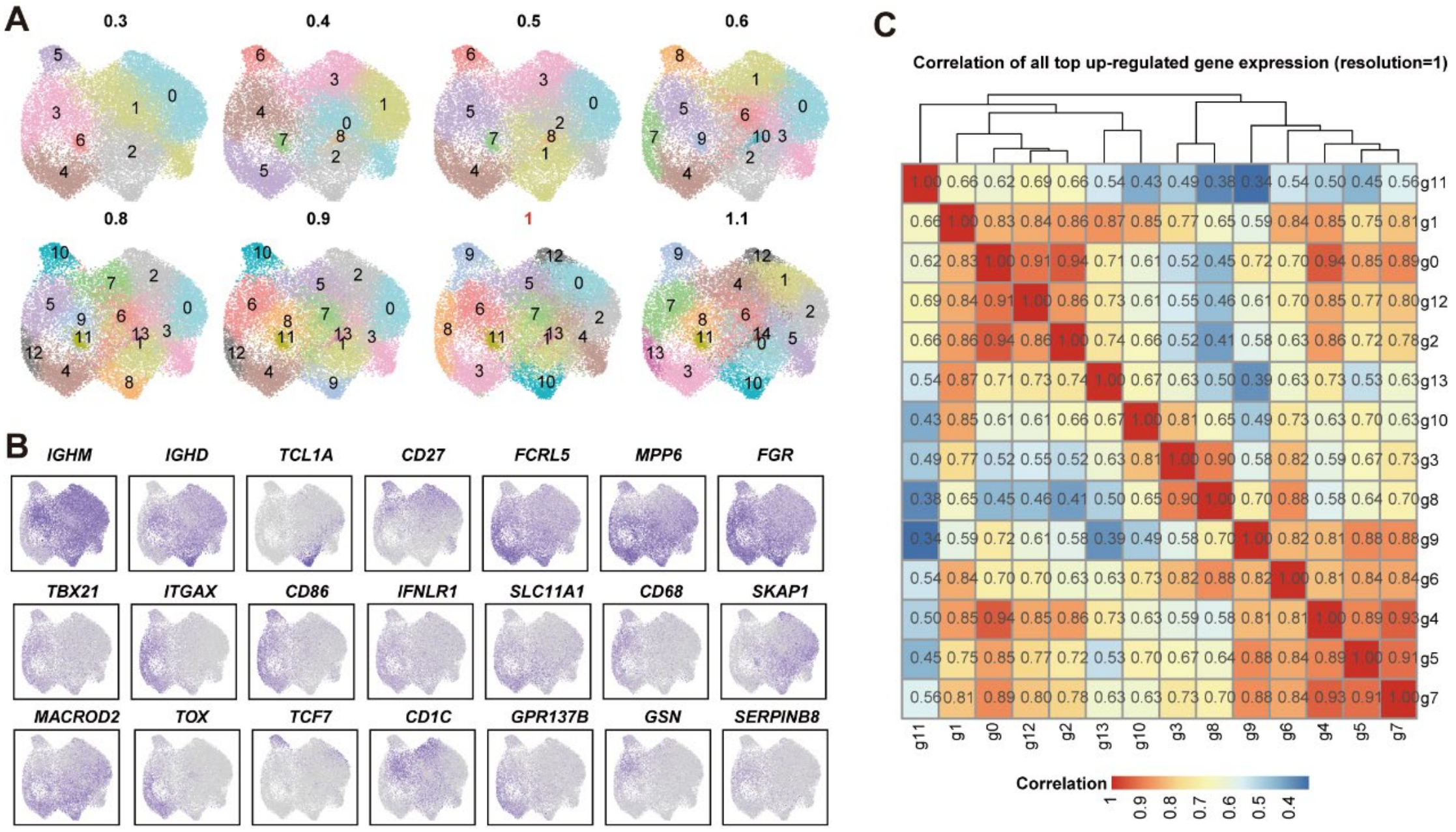


**Figure S14. Single-cell clustering of ABC clusters.**

(**A**) Single-cell clustering of all ABCs performed using the Louvain algorithm at multiple resolutions within the Seurat v5 workflow. Subplot titles indicate the corresponding resolutions. (**B**) UMAP projections showing gene expression of signature genes, including reference-curated markers and cluster-specific top upregulated genes. (**C**) Heatmap depicting the pairwise Pearson's correlation of average gene expression across clusters, computed using the top 50 upregulated genes per cluster ($\log_2$ fold change > 1, resolution = 1).

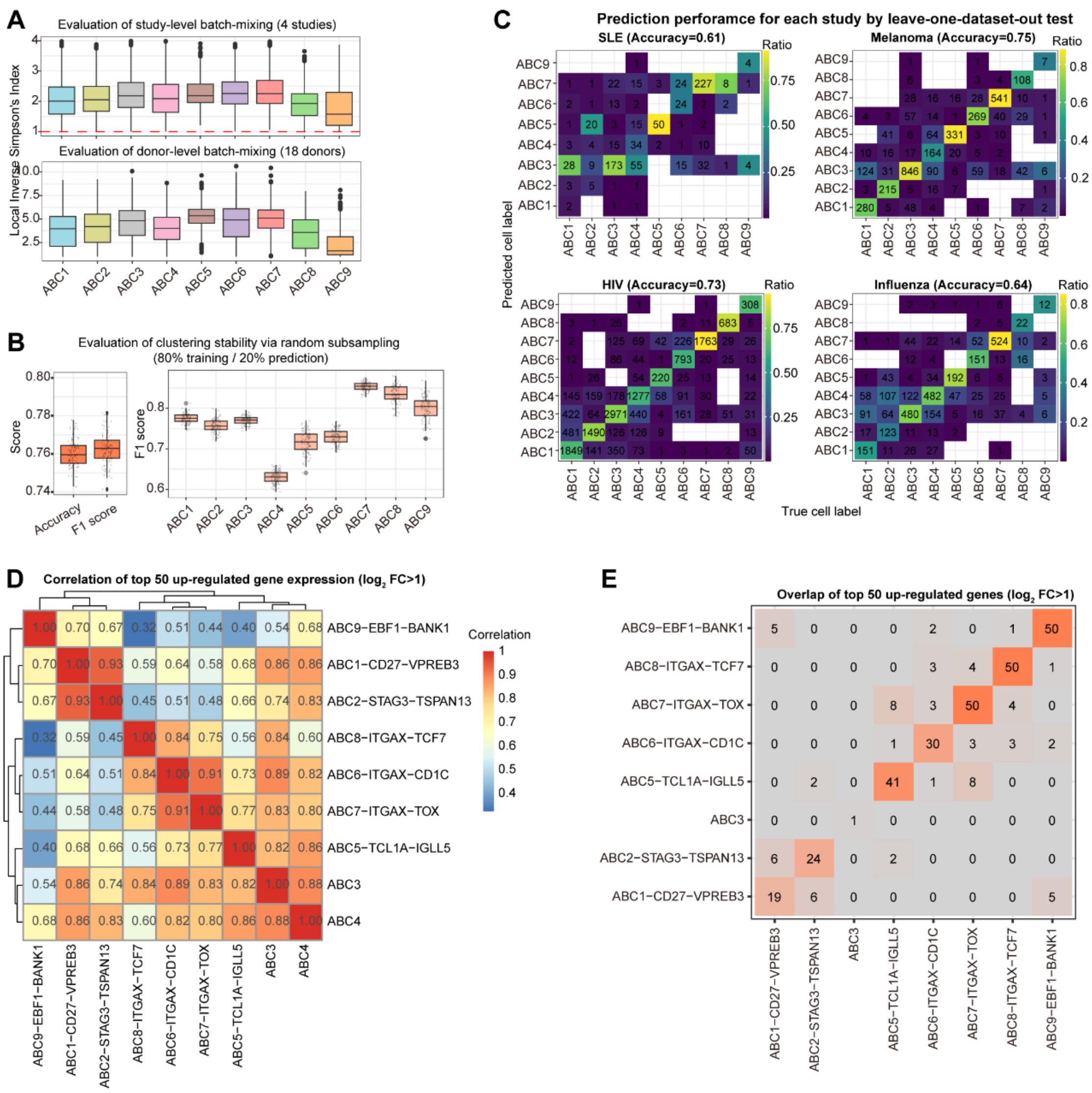


**Figure S15. Evaluation of ABC integration and clustering.**

(**A**) Boxplots illustrating the distribution of local inverse Simpsons indices, computed within the integrated RPCA space, across cell clusters at the study level (top) and donor level (bottom). (**B**) Boxplots showing the prediction performance across 100 testing sets (sampling 20% cells for 100 times). The left panel showing overall accuracies and F1 scores, while the right panel shows the distribution of F1 scores per cluster. (**C**) Heatmaps showing the confusion matrixes for each held-out dataset, generated using Seurats label transfer functions to predict cell labels for the held -out data. (**D**) Heatmap showing the correlation of average gene expression using merged up to top 50 up-regulated genes in each cluster. The number indicated the Pearson's correlation coefficient. (**E**) Overlap of up to top 50 up-regulated genes between diverse cell clusters by pairwise comparisons ($\log_2$ fold change>1). The diagonal represents the number of selected up-regulated genes based on ranking of adjusted *P* values and $\log_2$ fold changes.

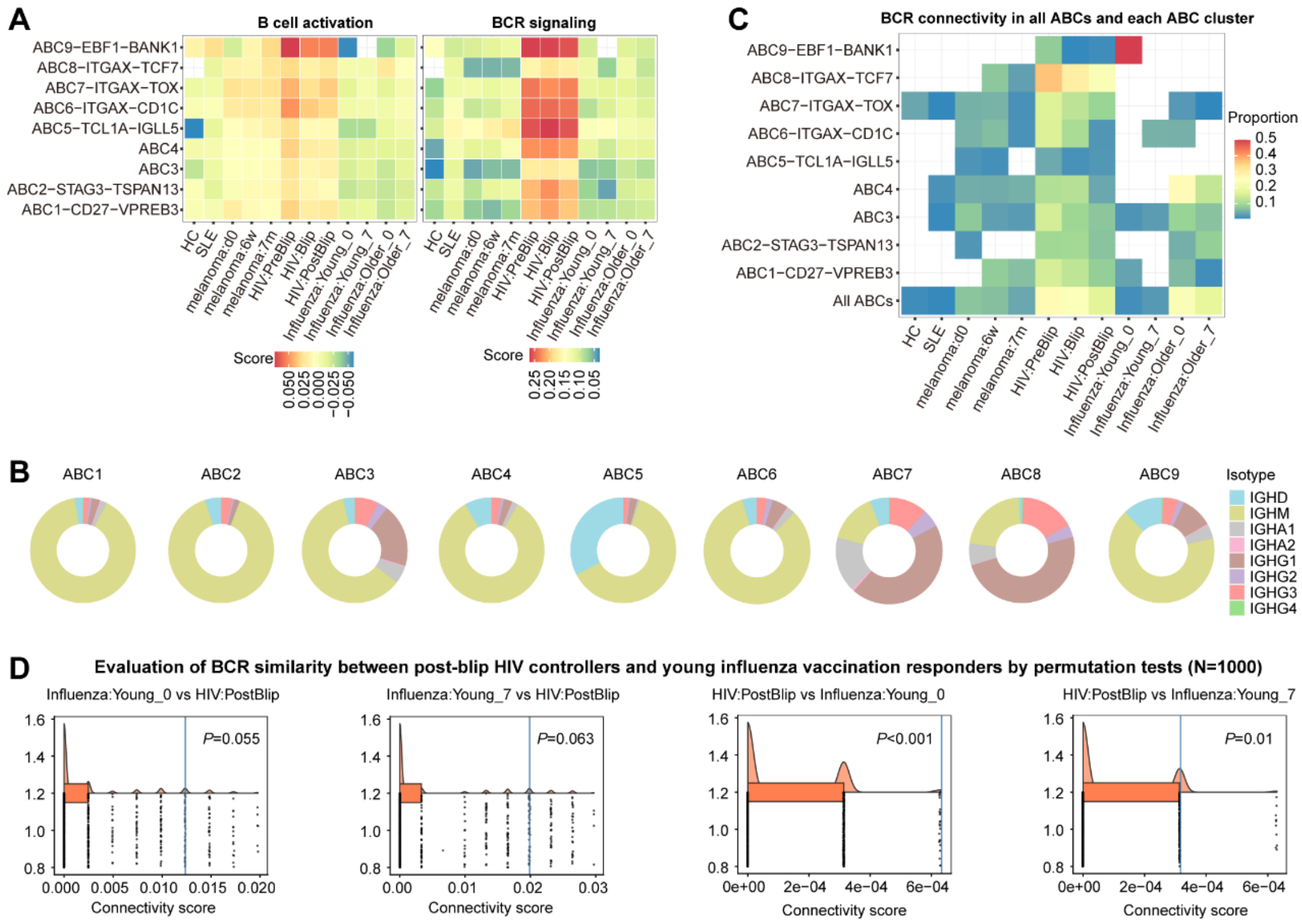


**Figure S16. Heterogeneity of ABCs across diverse disease contexts.**

(**A**) Distribution of average signature scores for different pathways across diverse cell clusters and phenotypes. (**B**) Distribution of BCR isotype compositions in each cell cluster. (**C**) Distribution of BCR connectivity in all B cells and diverse cell clusters across different phenotypes. (**D**) Raincloud plots showing the distribution of 1,000 permuted BCR connectivity scores between post-blip HIV patients and young influenza vaccine responders in four directional comparisons: pre-vaccination vs. post-blip HIV, post-vaccination vs. post-blip HIV, post-blip HIV vs pre-vaccination, and post-blip HIV vs. post-vaccination. Each point corresponds to a single permuted connectivity score. The *P* value was displayed on the top of each panel, defined as the fraction of permuted connectivity scores exceeding the observed score (indicated by the blue vertical line).

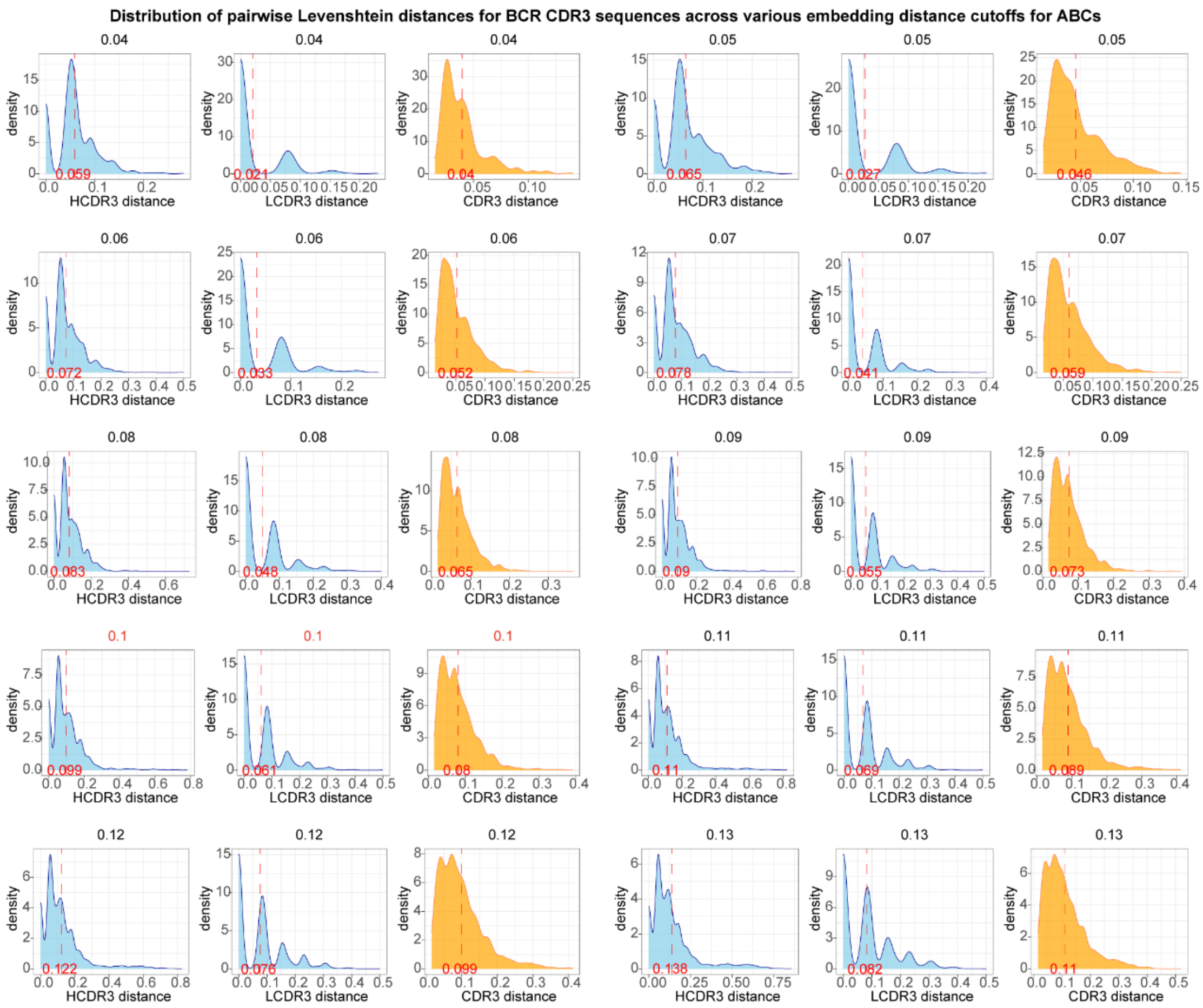


**Figure S17. Distribution of pairwise Levenshtein distances among BCR CDR3 sequences across various embedding distance cutoffs for ABCs.**

Density plots showing the distribution of pairwise Levenshtein distances among all BCR CDR3 sequences (heavy chain, light chain and paired) at varying embedding distance cutoffs for all ABCs. Each subplot title indicates the corresponding embedding distance cutoff, while the red dashed line marks the mean Levenshtein distance, with its value annotated adjacent to the line.
